# Melt electrowritten medium chain length polyhydroxyalkanoate cardiac patches for Post-MI cardiac regeneration

**DOI:** 10.1016/j.mtbio.2025.102256

**Published:** 2025-08-29

**Authors:** Qasim A. Majid, Pragati Pandey, Mohamed Bellahcene, Christopher L. Grigsby, Molly M. Stevens, Virpi Talman, Daniel J. Stuckey, Sian E. Harding, Ipsita Roy, Gábor Földes

**Affiliations:** a National Heart and Lung Institute, Faculty of Medicine, Imperial College London, London, W12 0NN, United Kingdom; b Heart and Vascular Center, Semmelweis University, Budapest, H1122, Hungary; c Drug Research Programme, Division of Pharmacology and Pharmacotherapy, Faculty of Pharmacy, University of Helsinki, Finland; d Department of Materials Science and Engineering, Faculty of Engineering, University of Sheffield, Sheffield, United Kingdom; e Insigneo Institute for in Silico Medicine, Pam Liversidge Building, Sir Robert Hadfield Building, University of Sheffield, United Kingdom; f Department of Medical Biochemistry and Biophysics, Karolinska Institute, Stockholm, 171 77, Sweden; g School of Pharmacy, Queens University Belfast, 97 Lisburn Road, Belfast, BT9 7BL, United Kingdom; h Department of Materials, Department of Bioengineering and Institute of Biomedical Engineering, Imperial College London, London, SW7 2AZ, United Kingdom; i Centre for Advanced Biomedical Imaging, Division of Medicine, University College London, London, WC1E 6DD, United Kingdom; j Research Centre for Integrative Physiology & Pharmacology, Institute of Biomedicine, University of Turku, Turku, Finland

**Keywords:** Melt electrowriting, Medium chain-length polyhydroxyalkanoates, Cardiac tissue engineering, Biomaterial scaffolds, Heart failure, Human pluripotent stem cell-derived cardiomyocytes, Human pluripotent stem cell-derived coronary microvascular-like endothelial cells

## Abstract

Human pluripotent stem cell-derived cardiomyocytes (hPSC-CMs) hold promise in averting the development of heart failure with reduced ejection fraction (HFrEF) following myocardial infarction (MI) by potentially regenerating the infarcted myocardium and restoring left ventricular contractility. However, challenges remain regarding the structural and functional maturation states of these cells, as well as their retention and integration into the myocardium. Here, we developed a novel three-dimensional cardiac patch and evaluated its potential to instigate cardiac regeneration. For the first time, melt electrowriting (MEW) was utilised to fabricate reproducible, structurally anisotropic, and handleable scaffolds from high molecular weight, medium chain-length polyhydroxyalkanoates (MCL-PHAs). These MEW-PHA scaffolds maintained hPSC-CMs, facilitating their rapid structural maturation and functional improvement in vitro. Different combinations of hPSC-derived cardiovascular cells were seeded onto the MEW-PHA scaffolds and stacked to create synchronously beating, multi-scaffold cardiac patches. These were well-accepted in a murine MI model without capsule formation. Notably, cardiac patches containing hPSC-derived cardiac microvascular-like endothelial cells (hPSC-CMVECs) initiated vascular regeneration within the infarcted myocardium. This novel advancement enabled the reproducible fabrication of high molecular weight MCL-PHA-based MEW cardiac patches that matured hPSC-CMs and promoted vascular regeneration, offering potential for future improvement in post-MI cardiac function through enhanced hPSC-CM retention.

## Graphical abstract

**Figure:**
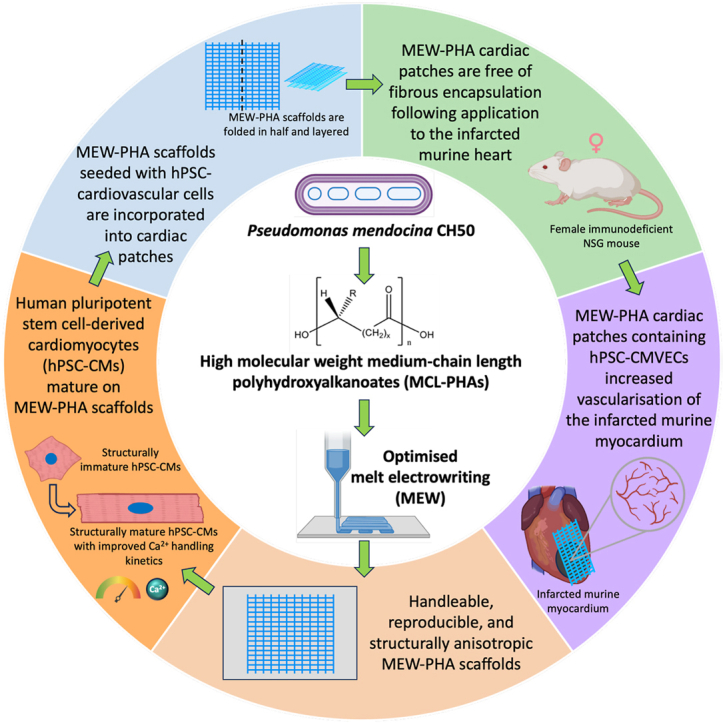

## 1 Introduction

The adult human heart is unable to instigate robust cardiac regeneration in response to injury. This is largely due to the post-mitotic nature of the contractile cardiomyocytes (CMs) [1]. Following ST-elevation myocardial infarction (STEMI, herein referred to as MI), the irreparable loss of CMs from within the myocardium of the left ventricle (LV) leads to the emergence of a large anterior infarct. The inability of residual adult CMs to readily proliferate and repopulate the infarct results in the formation of a fibrotic scar that perturbs cardiac function and drives progression towards heart failure with reduced ejection fraction (HFrEF) [2]. Current device-based or pharmacological therapies delay, but do not prevent, this injury cascade [3]. Given the five-year mortality rate for HFrEF patients exceeds that of the most common cancers [4], advanced therapeutic strategies that regenerate the infarcted LV and prevent post-MI cardiac functional decline are urgently required.

The epicardial application of biomaterial scaffolds to physically constrain the LV has been evaluated to prevent eccentric remodelling of the LV, a process that significantly contributes to post-MI functional decline [5]. However, in isolation, these scaffolds do not attenuate disease progression [6,7]. Cardiac tissue engineering (CTE) therefore aims to create regenerative cardiac patches by combining these scaffolds with cardiac cell therapies, themselves traditionally hindered by an inability to retain exogenous cells within the LV [8]. These patches can be derived from biodegradable biomaterials including natural human-derived biomaterials that excel in providing biocompatibility; although, this is often at the expense of mechanical properties [9]. Indeed, collagen is a major constituent of the myocardial extracellular matrix; however, collagen-derived hydrogels have an elastic modulus below 5 kPa [10], significantly below the tensile stiffness of the diastolic healthy adult myocardium (16 and 30 kPa for transverse and longitudinal fibre orientation, respectively) [10]. Myocardial stiffness also increases transiently during systole, where the myocardium is under tension [11], and becomes permanently elevated following MI [12].

The selection of a suitable biomaterial for CTE is multiparametric. In addition to being biocompatible, thus ensuring no further damage to the LV, the biomaterial must also be elastomeric to comply with the cyclic contractility of the myocardium. Mediumchain-length polyhydroxyalkanoates (MCL-PHAs) are an emerging family of natural and sustainable biopolymers produced via the nutrient-limited fermentation of bacteria. Despite their bacterial origin, MCL-PHAs display excellent biocompatibility both in vitro and in vivo [13]. This is in contrast to polycaprolactone (PCL), a well-investigated bioresorbable synthetic biopolymer routinely used in tissue engineering, where a thick fibrous capsule forms on the surface of PCL scaffolds four-weeks post administration [14]. This is indicative of a robust foreign body response (FBR) that is mediated by the infiltration and fusion of macrophages.

Alongside their superior biocompatibility, MCL-PHAs have mechanical properties well-suited for CTE applications [9]. Indeed, optimised fermentation conditions enable the production of MCL-PHAs with relatively high molecular weights (M_w_) [15], a parameter that heavily influences their mechanical properties. Whilst higher molecular weights are associated with increased stiffness [16], this may be beneficial in constraining the infarcted LV. More importantly, the relatively large monomeric units (6–14 carbon atoms) of MCL-PHAs both deter the formation of highly crystalline structures, known to induce an inflammatory response [17], and confer high elastomericity, thus these biopolymers are amenable to the cyclic nature of the contracting myocardium [13]. In addition, the long alkyl chains in the polymer backbone constitute a hydrophobic biopolymer that degrades gradually via surface erosion rather than via rapid bulk degradation [18]. The resultant degradation products are R-hydroxyalkanoic acids, natural metabolites found in the human body, that have a pKa higher than those emerging from the degradation of synthetic biomaterials such as poly(L-lactic acid) (PLLA). This lower acidity prevents further perturbation to the tissue parenchyma, once again highlighting the superior biocompatibility of MCL-PHAs relative to conventional synthetic biomaterials that have been investigated for CTE applications [19]. As such, MCL-PHAs were investigated in this study for the development of a novel cardiac patch.

Although biomaterial-based patches are intended to enhance the retention of exogenous cells relative to direct injection into the myocardium, several pre-clinical studies have shown that only a fraction of the exogenous human pluripotent stem cell-derived cardiomyocytes (hPSC-CMs) remain within the patch following administration [[20], [21], [22], [23]]. Nevertheless, this partial restoration of contractile tissue improves post-MI cardiac function. Enhancing the rate of hPSC-CM retention may yield superior functional benefits and could allow for future patches to be derived from fewer hPSC-CMs. This endeavour may be aided by incorporating cardiac endothelial cells (cECs) into the cardiac patch. Indeed, cECs are a critical component of the myocardium, with each CM surrounded by a dense network of ECs [24]. Crosstalk between CMs and ECs is essential to modulating CM maturity, contractility, and function [25,26], alongside generating the myocardial vasculature. Thus, their inclusion could facilitate the rapid formation of a primitive vascular network within the patch, which may anastomose with the host vasculature in the proximal healthy myocardium following administration, thereby sustaining the exogenous hPSC-CMs within the patch. This, coupled to synchronised bioresorption of the scaffold, could enable the longer-term viability, retention, and integration of hPSC-CMs within the myocardium where they could then contribute to contractility and prevent post-MI functional decline. Recent protocols outline the attainment of phenotypically stable human pluripotent stem cell-derived cardiac microvascular-like endothelial cells (hPSC-CMVECs) [27]. However, established small molecule differentiation protocols for the attainment of hPSC-CMs often yield cells with a structure, function, and gene expression profile akin to foetal CMs [[28], [29], [30]]. Intriguingly, hPSC-CMs respond to external stimuli including uniaxial mechanical stretch, as observed in collagen- or fibrin-derived engineered heart muscles/tissues (EHMs/EHTs) [31], and anisotropic cues in the form of grooves or micropatterns [32,33], leading to their adoption of a more mature phenotype with characteristics of adult CMs.

The precise design and fabrication of biomaterial scaffolds allows for the integration of defined anisotropic cues, which could enable a patch derived from these scaffolds to simultaneously mature and deliver hPSC-CMs to the infarcted LV. Melt electrowriting (MEW), also referred to as melt electrospinning writing, is a relatively novel three-dimensional (3D) additive manufacturing process that combines the principles of conventional solution electrospinning with that of 3D printing [34]. To this end, a polymer melt undergoing pressure-mediated extrusion interacts with an electrical field to generate a polymer jet. In contrast to conventional solution electrospinning, the controlled, 3D deposition of this jet enables the fabrication of handleable and reproducible scaffolds with a pre-defined architecture. MCL-PHAs exhibit thermal properties conducive to the fabrication of scaffolds via such melt-based extrusion techniques [35]. However, owing to their high M_w_, MCL-PHAs have a higher viscosity [36]. Conventional electrospinning surmounts this by modulating the solvent concentration; in contrast, MEW forgoes these cytotoxic solvents. Whilst this is advantageous to the clinical translation of a CTE emerging from this technique, and allows for the precise, pre-defined geometry of the emerging scaffold to be achieved, the need to modulate the melt polymer viscosity has thus far largely limited MEW to the use of low M_w_ (<100 kDa) variants of PCL that intrinsically have a lower viscosity [37].

This study outlines, for the first time, the multi-step optimisation of MEW using the high M_w_ (281.16 kDa) MCL-PHA poly(3-hydroxyoctonate-*co*-3-hydroxydeconate) [P(3HO-*co*-3HD)] to attain reproducible, structurally anisotropic, and handleable MEW-PHA scaffolds. Thereafter, the in vitro biocompatibility of these scaffolds was assessed by investigating their capacity to sustain and mature different combinations of hPSC-derived cardiovascular cells (hPSC-CMs and hPSC-CMVECs). Subsequently, these scaffolds were stacked to generate synchronously beating, multi-scaffold cardiac patches. The first iteration of these patches was assessed in an immunodeficient mouse model of MI, where their in vivo biocompatibility was assessed, in addition to their potential to attenuate eccentric remodelling of the infarcted LV, the ability of the patch and the hPSC-CMVECs to enhance retention of hPSC-CMs post-administration, and the effect of this on post-MI cardiac function. This study therefore presents critical and novel advancements, significantly enhancing the applications of MEW in CTE, as well as in broader tissue engineering and regenerative medicine strategies beyond the cardiovascular field. Further, our in vivo investigation provides proof of principle for the biocompatibility of MCL-PHA-derived cardiac patches, whilst also offering insight into the limited impact of vascular regeneration alone on post-MI cardiac function in the absence of substantive contractile tissue regeneration.

## 2 Materials and methods

### 2.1 Experimental design

The primary objectives of this study focused on the design and assessment of a novel CTE solution to regenerate the infarcted LV. To achieve this, we firstly evaluated the potential to process three high M_w_ MCL-PHAs using MEW, for the attainment of reproducible and structurally anisotropic scaffolds that could be handled with ease. Thereafter, in vitro assessment of the hPSC-cardiovascular cells seeded on the MEW-PHA scaffolds was conducted via phenotypic characterisation and calcium imaging. Subsequently, different compositions of the cardiac patch were derived through the stacking of the individual scaffolds. To ascertain which component(s) of the patch were imperative to regeneration – mechanical support, contractile tissue, vasculature, – acellular-, hPSC-CM monoculture-, hPSC-CMVEC monoculture-, and co-culture-patches were evaluated in immunodeficient mice following induction of MI via permanent ligation of the left anterior descending (LAD) coronary artery. Although this model does not mimic the clinical goal in which the occluded coronary artery should be reperfused in a timely manner via percutaneous coronary intervention (PCI), it consistently and reproducibly creates large transmural infarcts that lead to eccentric remodelling of the LV and a significant reduction in the LV ejection fraction (LVEF) [38]. This therefore enables subtle improvements in this functional parameter to be determined and was thus selected for our study. To minimise the effects of the hosts adaptive immune response and to circumvent the need for immunosuppression, immunodeficient NOD scid gamma (NSG) mice were utilised within. Cardiac MRI and histological examination were performed 4-weeks after concurrent MI-induction and patch administration to ascertain cardiac structure function, as well as cellular composition of the LV, respectively. In vivo experiments and downstream analysis were conducted in a blinded and randomised fashion.

### 2.2 Production and characterisation of MCL-PHAs

Poly-3-hydroxyoctanoate [P(3HO)], Poly(3-hydroxyoctanoate-*co*-3-hydroxydecanoate) [P(3HO-*co*-3HD)], and Poly(3-hydroxyoctanoate-*co*-3-hydroxydecanoate-*co*-3-hydroxydodecanoate) [P(3HO-*co*-3HD-*co*-3HDD)] were produced using the nitrogen-limited batch fermentation of *Pseudomonas mendocina* CH50 in the presence of either sodium octanoate, glucose, or coconut oil, respectively, as previously described [15,39,40]. Carbon 13 (^13^C) NMR, differential scanning calorimetry (DSC), and gel permeation chromatography (GPC) were conducted as previously outlined [41] to determine the chemical structure, thermal properties, and M_w_ of the generated polymers, respectively.

### 2.3 Production of MCL-PHA solvent cast films

MCL-PHAs were dissolved in chloroform to generate a 5 % (w/v) chloroform-polymer solution, that was cast into a glass petri dish and placed within a fume hood for one week to enable evaporation of the chloroform.

### 2.4 Assessment of the MCL-PHA MEW polymer jet lag

Photographs of the MCL-PHA polymer jet were taken from outside the glass cabinet of the MEW system as the XY stage moved at 5 mm/s. Images were calibrated and subsequently analysed in FIJI. The distance between the point directly below the spinneret and the point at which the fibre was deposited onto the XY stage was calculated as the MCL-PHA jet lag length. A representative frame was analysed per video with three videos analysed per MCL-PHA.

### 2.5 Computer aided design (CAD) of the MEW-PHA scaffolds

MEW-PHA scaffolds were designed in the SEL Program Generator Dispenser software (IAI corporation) from which a points table and scaffold programme file were generated. The translation speed, the cycle count (determining the number of layers in each scaffold), and the acceleration and deceleration of the XY stage, were specified in this software. The optimised MEW-PHA scaffolds were comprised of an initial 1 cm^2^ outer box from which 0.5 cm ‘edge loops’ projected vertically and horizontally (Fig. S1Ai). Fibres were then designed to be deposited in the x-axis followed by the y-axis, extending 0.5 cm beyond the outer box on each side (Fig. S1B and C). The distance between neighbouring fibres was dependent upon the desired pore dimensions. A ‘printing pause’ step was specified by instructing the XY stage to return to its origin where it was held for 1 min prior to initiating a further cycle (Fig. S1C). The points table and the scaffold programme file were loaded into the ‘PC interface software for XSEL’ (IAI corporation) from which the MEW system was controlled.

### 2.6 Fabrication of MEW-PHA scaffolds

MEW-PHA scaffolds were fabricated via the ‘MELT’ MEW system (Spraybase) that comprised of: a syringe barrel and spinneret (0.25–0.80 mm, E3D) placed into the heating head to melt the MCL-PHA, a nitrogen gas line to extrude the molten polymer through the spinneret, an electrified XY stage to generate a polymer jet and control its 3D deposition, and a movable z-axis to allow for multi-layered scaffolds. 1g of MCL-PHA was cut into subcentimeter pellets and loaded into the syringe barrel where it was heated to 95 °C for 15 min. Scaffolds were printed onto glass microscope slides (Sigma-Aldrich) affixed to the XY stage. The z-axis was lowered via the XSEL software to bring the spinneret to within 1 cm of the slide. A stable polymer jet was established with 50 kPa extrusion pressure and 15 kV voltage after which writing of the loaded scaffold design was initiated at a translation speed of 5 mm/s with acceleration and deceleration rates of 0.01 G and 1.5 G, respectively. Extrusion pressure and voltage were switched off during the printing pause steps and thereafter, returned to the system for the next cycle that was initiated upon re-establishment of a stable polymer jet. The subsequent cycle was deposited upon the underlying layer. Upon completion of the writing programme, the glass microscope slide onto which the MEW-PHA scaffolds were fabricated were removed from the XY stage and placed at −20 °C for 1 min. MEW-PHA scaffolds were detached from the glass slide using ice-cold 100 % ethanol and a metal blade, which was also employed to cut the edge loops and fibre extensions to attain the final 1 cm^2^ MEW-PHA scaffold.

### 2.7 Evaluation of the structural reproducibility of MEW-PHA scaffolds

Brightfield microscope images of MEW-PHA scaffolds were analysed in FIJI by manually drawing a calibrated line across the length and width of a scaffold pore from which the diagonal length and aspect ratio (AR) of the pore could be determined. The interquartile range (IQR) of the diagonal length was calculated to ascertain the reproducibility of the printing parameters by evaluating the degree of variation in pore size. A tolerance of 10 % between the predicted and actual calculated diagonal length of the pores was applied to assess the achievable resolution of MEW with MCL-PHAs. Thirty pores were analysed per scaffold and three scaffolds were analysed per writing condition.

### 2.8 Scanning electron microscopy (SEM) of MEW-PHA scaffolds

MEW-PHA scaffolds were mounted onto SEM pin stubs using conductive carbon adhesive tape and sputter coated with 15 nm chromium for 15 min using a Q150T Plus turbomolecular pumped coater (Quorum Technologies). Scaffolds were visualised via the LEO Gemini 1525 field emission gun scanning electron microscope (FEG SEM) (ZEISS) at the Department of Material Science, Imperial College London, UK.

### 2.9 Tensile testing of the MEW-PHA scaffolds and MCL-PHA strips

The scaffold design was upscaled to 5.0 mm × 2.3 mm, whereas MCL-PHA strips of the same dimensions were cut from solvent cast films. Scaffolds and strips were each loaded into the 5940 Series Universal Testing System (Instron) and their thickness was determined via a digital calliper. Thereafter, a defined programme of elongation (10 mm/min) was initiated to generate stress-strain curves from which the -ultimate tensile strength (σ_μ_), -elastic modulus (E), and apparent-elongation at break (ε_b_) were calculated.

### 2.10 Cardiovascular differentiation of human pluripotent stem cells

hPSC-CMs were derived from H7 human embryonic stem cells (hESCs, WiCell) via a previously reported small molecule, Wnt/β-catenin modulation protocol [[42], [43], [44]]. hPSC-CMs were maintained in RPMI-1640 media (Sigma-Aldrich) containing B-27^TM^ supplement (Gibco, RB^+^) and used for experimentation 30 days (day 30) after the initiation of the differentiation protocol unless otherwise stated. H7-derived hPSC-CMVECs were attained via a recently established 3D, growth-factor based differentiation protocol and cultured in EGM2 media (Lonza) containing 50 ng/ml VEGF-A (Peprotech, EGMV) [27]. The hPSC-CMs and hPSC-CMVECs are collectively referred to as hPSC-cardiovascular cells.

### 2.11 Culturing hPSC-cardiovascular cells on MEW-PHA scaffolds

MEW-PHA scaffolds were sterilised in 100 % ethanol for 1 h and washed in sterile phosphate-buffered saline (PBS) for a further 1 h to remove traces of cytotoxic ethanol. A single scaffold was placed into an individual well of an 8 well chamber slide (Ibidi) that had previously been coated with 0.5 % w/v Pluronic® F-127 (Sigma-Aldrich) to prevent cells from adhering to the surface of the slide [45]. A single side of a MEW-PHA scaffold was seeded with hPSC-cardiovascular cells for 12 h at 37 °C. Owing to their limited proliferation potential, hPSC-CMs were seeded to confluency on MEW-PHA scaffolds by adding 7.5 × 10^5^ hPSC-CMs (CM monoculture scaffold). As hPSC-CMVECs readily proliferate, they were seeded at a lower density of 5.0 × 10^5^ hPSC-CMVECs (EC monoculture scaffold). A 3:1 ratio of hPSC-CMs: hPSC-CMVECs was also established for the co-culture scaffolds. Following seeding, scaffolds were transferred to a 24 well plate and maintained for up to two weeks in their respective complete media or, in the case of co-cultured scaffolds, a 50 %:50 % composition of RB^+^ and EGMV. MCL-PHA solvent cast films were similarly sterilised and placed into a 24 well plate where they were seeded with 125,000 hPSC-CMs/cm^2^ and cultured for up to 1 month. Age-matched controls were attained by seeding 10,000 hPSC-CMs into a 10 mm micro-well of a 35 mm glass bottom MatTek dish (MatTek corporation) that had been pre-treated with bovine fibronectin (Sigma-Aldrich) for 1 h.

### 2.12 Optical mapping of intracellular calcium transients

The calcium indicator Fluo-4-AM (Invitrogen) was diluted in 20 % w/v Pluronic® F-127 (Sigma-Aldrich) and added to pre-heated (37 °C) Tyrode's solution. Cell-laden MEW-PHA scaffolds were washed with PBS and transferred to an empty MatTek dish where they were incubated in Fluo-4-AM-containing Tyrode's solution for 25 min at 37 °C. MatTek control dishes were similarly washed and incubated. Thereafter, pre-heated Tyrode's solution was introduced to both the control and scaffold-containing MatTek dishes that were then placed into the preheated chamber of a Axio Observer widefield microscope (Zeiss). Carbon electrodes were introduced into the Tyrode's solution and 1Hz, 20V field stimulation was applied. Four second video recordings were acquired at dimensions of 200 x 2048 pixels to ensure a frame rate of 500 frames per second. Intensity time traces were generated from these videos via the Time Series Analyser FIJI plugin [46] that were then analysed via the Clampfit module of the pCLAMP 10.7 software (Molecular Devices) to determine time-to-peak transient (TTP), and time-to-50 %-, −75 %-, and −90 %-decay of the calcium transient.

### 2.13 Generation of MEW-PHA cardiac patches

Patches were derived from two-identical MEW-PHA scaffolds that, following one week of culture in isolation, were stacked upon one another by firstly folding each scaffold in half lengthwise and then placing upon each other ensuring that the cell-seeded sides were in direct contact between the two scaffolds (Fig. S6). A non-resorbable 9-0 Ethilon nylon suture was applied to either side of the patch to maintain the position of the individual scaffolds (Fig. 3F, S6). The patch was then placed in a 12-well tissue culture plastic (TCP) plate for a second week of in vitro culture. Each complete patch was therefore seeded with the following cellular compositions: acellular, no cells; hPSC-CM monoculture, 1.5 × 10^6^ hPSC-CMs; hPSC-CMVEC monoculture, 1.0 × 10^6^ hPSC-CMVECs; and co-culture, 1.5 × 10^6^ hPSC-CMs with 0.5 × 10^6^ hPSC-CMVECs.

### 2.14 Immunofluorescent staining

Upon reaching the pre-defined experimental end point, cell-laden MEW-PHA scaffolds or MatTek control dishes (herein: fibronectin-coated TCP) were washed with PBS and fixed in 4 % paraformaldehyde (Thermo Fisher Scientific) for 15 min at room temperature (RT). The fixed samples were permeabilised in 0.2 % Triton X-100 (Thermo Fisher Scientific) PBS solution for 10 min and subsequently blocked in 4 % foetal bovine serum (FBS)-PBS solution for 1 h. Primary antibodies were dissolved in 3 % bovine serum albumin (BSA) and incubated with the samples for 1 h at RT. Samples were washed 3 times in PBS and incubated with the appropriate secondary antibody for 45 min at RT before counterstaining with the nuclear dye, Hoechst 33342 (Abcam) for 15 min at RT. The antibodies employed in this study were used at the following concentrations: anti-α-actinin (1:200, #A7811, Sigma-Aldrich), anti-cardiac troponin T (1:200, #ab45932, Abcam), anti-CD31 (1:100, #558068, BD Biosciences), anti-FSP1 (1:100, #07–2274, Sigma-Aldrich), anti-Ki67 (1:100, #ab833, Abcam), Alexa Fluor (AF) 488 donkey anti-mouse (1:400, #A21202, Thermo Fisher Scientific), and AF 546 donkey anti-rabbit (1:400, #A10040, Thermo Fisher Scientific).

### 2.15 Phenotypic characterisation of immunofluorescent stained hPSC-CMs

Anti-α-actinin demarcates the Z-line of the hPSC-CM sarcomere and was used to determine sarcomere length in FIJI by drawing a perpendicular line across seven Z-lines within a single hPSC-CM. The ‘maxima intensity difference’ value attained by the line profile tool revealed the distance between neighbouring Z-lines and thus determined the length of the sarcomeres. The maxima difference value across the seven Z-lines was averaged to attain the average sarcomere length. Anti-α-actinin staining was also utilised to ascertain the alignment of hPSC-CMs. As each sample was indiscriminately placed onto the microscope at different angles, the images were rotated in a non-biased manner via the ‘horizontal alignment’ tool of the Orientation J plugin [47] to attain comparable images. The directionality analysis tool was then used as previous [48] to determine the number of pixels arising from the sarcomeric Z-lines present at angles between −90° and 90° with measurements taken equally every 2.02° to attain 90 measurements. A ‘half maximum’ value was ascertained by identifying the maximum count value (a.u.) and dividing this by two to determine alignment. Aligned samples had higher values relative to poorly aligned samples that had pixels in many angles. The aspect ratio of hPSC-CMs was determined by drawing around an individual hPSC-CM and determining the shortest and longest dimensions via the ‘find minimum and maximum feret’ tool. The aspect ratio was calculated by dividing the maximum feret value by the minimum value. A minimum of 30 hPSC-CMs were analysed per N for each phenotypic analysis.

### 2.16 Myocardial infarction induction and application of the MEW-PHA cardiac patches

Eight-to ten-week-old, female NSG mice (Charles River) were utilised for this study. All animal experiments conformed to the UK Animals (Scientific Procedures) Act, 1986, amended with Regulations 2012 to transpose European Directive 2010/63/EU. The procedure was performed under aseptic conditions. After induction anaesthesia (4 % isoflurane; 1 l/min O_2_), mice received 0.1 mg/kg of buprenorphine by subcutaneous injection (Vetergesic, Alstoe Animal Health). Eye ointment was applied, and mice were placed in a supine position where they were intubated and ventilated. A tidal volume of 250 μl and a respiratory rate of 150 breaths min^−1^ (Hugo-SachsMiniVent type 845; Harvard Apparatus) was used to deliver 2 % isoflurane; 0.5 l/min O_2_. The chest was shaved, and a povidone iodine skin disinfectant was applied. A film dressing was then placed over the chest to prevent fur from entering the incision. The skin and underlying muscle layers were blunt dissected, and the pericardium was removed following a left thoracotomy in the fourth intercostal space. A 6-0 polypropylene suture was used to permanently ligate the LAD coronary artery. The ligature was consistently positioned ∼2–3 mm below the atrio-ventricular junction. Immediately following LAD ligation, the MEW-PHA cardiac patch was placed onto the LV in all groups except the sham operated and MI-only animals and secured in place by passing a single 9-0 PROLENE® polypropylene suture through the myocardium, at the top and bottom of the patch. In sham operated animals, the suture was passed under the LAD; however, it was not ligated and thereafter removed. Following intervention, the separated ribs, muscle, and skin layers were closed with appropriate sutures at which point the wound was disinfected. The isoflurane anaesthesia was turned off; however, mechanical ventilation continued until the mouse regained a gag reflex. Operated mice were given 0.5 ml saline via subcutaneous injection to counter dehydration and were placed into a heated recovery chamber for 20 min. Following visual assessment, the operated mice were returned to a normal holding cage with supplemental heat and food-water solution placed at floor level. Adequate post-operative care and monitoring were provided.

Real time monitoring of ECG, heart rate, and core body temperature during surgery was conducted using the rodent surgical monitor platform (Indus Instruments). Consistency of the surgical MI model was ensured by the application of predefined surgical inclusion criteria: clear ST segment elevation after LAD ligation, distinct myocardial blanching, akinesis of the myocardium below the ligature, and core body temperature within 36–38 °C. Body temperature was maintained at 37.0 ± 0.5 °C throughout the procedure. This strict temperature control is imperative given the known cardioprotective effect of mild hypothermia (34–36 °C) [49].

### 2.17 Assessment of cardiac function via cardiac MRI

Cardiac imaging was performed as described previously [50,51]. Four weeks after surgical intervention, mice were firstly anaesthetised and maintained with 2 % isoflurane in oxygen prior to imaging via a 9.4T MRI system (Agilent) and a 38 mm quadrature driven birdcage RF coil (Rapid Biomedical).

Cardiac function was quantified using cine imaging in the short axis view using a stack of eight to ten slices to cover the whole LV. A cardiac and respiratory-gated gradient echo sequence was used with the following parameters, echo time: 1.18 ms, repetition time: 5 ms, flip angle: 15°, slice thickness: 1 mm, field-of-view (FOV): 25.6 × 25.6 mm, matrix size: 128 × 128, number of signal averages: 2. The analysis of cardiac function from cine images was performed using FIJI. End diastolic volume (EDV) and end systolic volume (ESV) were ascertained from the segmentation of either the LV endocardium in all end-diastole slices or all end-systole slices, respectively. LVEF was calculated from the stroke volume (SV), itself derived from the EDV and ESV. Cardiac output (CO) was calculated from the SV and heart rate as per conventional physiological calculations.

Infarct size was quantified using late gadolinium enhancement MRI (LGE-MRI). Gadopentetate dimeglumine (Gd-DTPA) was administered via an intraperitoneal injection as a single bolus of 0.5 mmol kg^−1^. Images were acquired using a cardiac and respiratory-gated multi-slice inversion-recovery gradient echo sequence with a single inversion time selected from a look-locker scout to null the remote myocardium (typically 250–350 ms). Sequential acquisitions of eight to ten short-axis slices to cover the entire left ventricle were performed using the following parameters: echo-time: 3.04 ms, inter-slice repetition time: 1.11 ms, inter-inversion repetition time: 800–1000 ms dependent on respiratory rate, flip angle: 90°, slice thickness: 1.0 mm, FOV: 25.6 × 25.6 mm, and matrix size: 192 × 192.

Images were analysed using FIJI with semiautomated LV mass and infarct mass. LV mass was measured by semiautomated segmentation of the myocardial area in all slices of the inversion recovery acquisition. LGE-MRI enhanced areas (hyperintense region) designated infarct areas. The region of enhancement was defined as any with a signal intensity 3 standard deviations above remote myocardium. Infarct mass measurement was performed in every slice by visual assessment and manual tracing of the enhanced area in LGE-MRI. The LV and infarcted mass were calculated as segmented LV and infarcted area multiplied by the slice thickness (1 mm) and the specific gravity of the myocardium (1.05). The percentage of infarct mass was calculated as infarct mass/total LV mass. Percentage infarct area was calculated from the surface area of the infarct/total LV surface area – infarct area and represents a more accurate quantification of infarct size than infarct volume in chronic mouse infarcts where the LV wall has thinned to such a degree that volume measurements can be inconsistent.

### 2.18 Histological assessment of mouse hearts

Following cardiac MRI, mice were humanely culled using the schedule 1 neck dislocation method and hearts were perfused with PBS followed by 4 % PFA. Thereafter, the heart was excised and placed into a 4 % PFA solution in PBS for 24 h at RT. The fixed hearts were transferred to a 70 % ethanol solution for a further 24 h followed by PBS for longer term storage at 4 °C. Hearts were placed onto a mouse heart slicer matrix (Zivic Instruments) and sectioned into 3 mm serial sections that were embedded in paraffin and further sectioned into 5 μm sections that were deposited on slides.

For immunofluorescent staining, the sections were first deparaffinised by immersing the sections twice in Neo-clear (Sigma-Aldrich) for 10 min each and gradually rehydrated by immersion in 100 % ethanol twice for 5 min each, followed by 95 %, 70 %, and 30 % ethanol for 5 min each. Sections were then placed in distilled water for 5 min and antigen retrieval was performed by boiling sections in 0.1 M citrate buffer (Sigma-Aldrich) for 20 min in a microwave. Sections were subsequently permeabilised by immersion in 0.1 % Triton/PBS (Sigma-Aldrich) for 10 min and blocked in 1 % BSA/10 % normal goat serum (Thermo Fisher Scientific) for 1 h at RT. The blocking solution was then replaced with primary antibodies at the following concentrations for 16 h at 4 °C: isolectin GS-IB4 (1:1000, #I21411, Thermo Fisher Scientific) and anti-cardiac troponin T (1:500, #ab10214, Abcam). Thereafter, sections were washed 3 times in PBS for 10 min and incubated with AF 680 goat anti-mouse (1:400, #115-625-146, Jackson ImmunoResearch Laboratories) for 16 h at 4 °C. On day 3 of the staining protocol, sections were washed three times in PBS for 10 min each and stained with 4′,6-diamidino-2-phenylindole (DAPI) (Thermo Fisher Scientific) for 20 min at RT followed by a round of three PBS washes for 10 min each. Slides were then mounted using 2 drops of ProLong Gold Antifade Reagent (Thermo Fisher Scientific) and stored in the dark at 4 °C until imaged.

For haematoxylin and eosin staining, sections were deparaffinised and hydrated as described above and incubated in Mayer's Haematoxylin solution (Sigma-Aldrich) for 2 min. Sections were washed in running tap water for 15 min followed by incubation in Eosin Y solution (Sigma-Aldrich) for 2 min. Sections were then washed with PBS for 5 min and dehydrated in an increasing concentration of ethanol from 30 %, 70 %, 95 %, and 100 % for 5 min each. Finally, slides were mounted with Dibutyl Phthalate Xylene (DPX) (Sigma-Aldrich) and imaged. Masson's trichrome staining was conducted using the Masson's Trichrome Staining Kit (Agilent Dako) according to manufacturer's instructions.

For 3,3′-Diaminobenzidine (DAB) staining, sections were again deparaffinised and hydrated as described above prior to incubation in methanolic hydrogen peroxide for 10 min to block endogenous peroxidases. Thereafter, citrate antigen retrieval was conducted by placing sections into pre-heated citric acid buffer (10 mM citric acid, 0.05 % Tween 20, pH 6.0 using 1N HCL) and boiling for 20 min. Slides were cooled to RT in PBS and samples were permeabilised in 0.1 % Triton X:PBS solution for 10 min. Slides were washed in PBS and rinsed in tap water for 5 min. A PAP pen was used to draw a hydrophobic barrier around the sample to retain solutions within this area. A blocking solution derived from 10 % normal goat serum in 1 % BSA: Gold buffer solution was applied to the samples for 1 h. Following blocking, samples were incubated with the primary antibody anti-Iba1 (1:200, #ab5076, Abcam) that had diluted in 1 % BSA, and incubated for 16 h at 4 °C. Sections were then washed three times in 0.1 % Triton/PBS for 10 min each and subsequently incubated with the biotinylated secondary antibody biotin donkey anti-goat (1:500, #ab6884, Abcam) prepared in PBS without serum. DAB staining was conducted using the ABC-HRP kit and DAB substrate kit (Vector Laboratories) according to manufacturer's instructions.

To assess retention of exogenous hPSC-cardiovascular cells within the patch, patches were cropped manually, and a custom pipeline was created within the CellProfiler image analysis software to firstly separate the channels and thereafter, count the total number of nuclei within the patch alongside the number of nuclei positive for either isolectin or cardiac troponin T. This allowed for the calculation of the percentage of retained hPSC-CMVECs and hPSC-CMs, respectively. Capillary density was calculated by firstly drawing around the infarct zone and the border zone to calculate their area. Capillary structures positive for isolectin were then counted manually and presented as capillaries/mm^2^.

### 2.19 Statistical analysis

Statistical analysis was conducted in Prism 10 (GraphPad) and RStudio. All experiments were conducted to a minimum of three repeats (N), unless otherwise stipulated. MEW studies utilised MCL-PHAs generated from a minimum of three separate fermentations, whilst in vitro cellular experiments utilised hPSC-CMs and hPSC-CMVECs arising from at least three separate stem cell differentiations (minimum of three biological repeats). Where appropriate, individual data points are shown, with each point representing a separate experiment conducted using hPSC-cardiovascular cells from distinct differentiations. Values are presented as mean ± standard deviation (S.D.) unless otherwise stated. A Grubbs' test with an alpha value of 0.05 was first used to identify outliers which were subsequently removed from the dataset. The distribution of data was then assessed via a Shapiro-wilk normality test. For parametric data, a Student's t-test was used to calculate significance between two groups whilst comparison between more than two groups utilised one-way or two-way ANOVAs (analysis of variance) followed by an appropriate post-hoc test (Bonferroni's, Tukey's, or Dunnet T3, as indicated in the figure legend). For non-parametric data, a Kruskal-Wallis test was performed, followed by a Dunn's post-hoc test. A P-value of less than 0.05 was considered significant with statistical significance indicated as follows: ∗P < 0.05, ∗∗P < 0.01, ∗∗∗P < 0.001, ∗∗∗∗P < 0.0001. P-values greater than 0.05 were deemed non-significant (ns).

## 3 Results

### 3.1 Optimised MEW allowed for the fabrication of structurally anisotropic scaffolds from MCL-PHAs

**Fig. 1:**
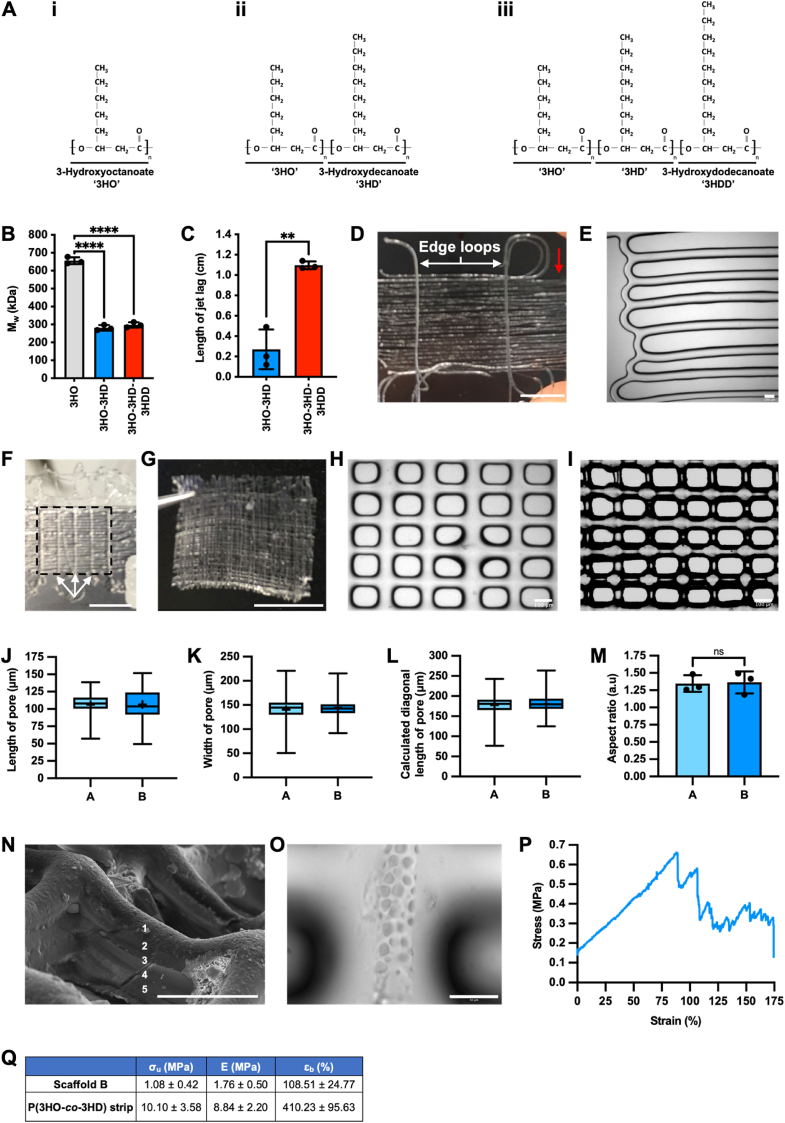
Optimisation and fabrication of MEW-PHA scaffolds: (**A**) Monomeric units of (**i**) Poly(3-hydroxyoctanoate) (‘3HO’), (**ii**) Poly(3-hydroxyoctanoate-*co*-3-hydroxydecanoate) (‘3HO-3HD’), and (**iii**) Poly(3-hydroxyoctanoate-*co*-3-hydroxydecanoate-*co*-3-hydroxydodecanoate) (‘3HO-3HD-3HDD’), as determined via ^13^C NMR. (**B**) Molecular weight (M_w_) of P(3HO) (grey bar), P(3HO-*co*-3HD) (blue bar), and P(3HO-*co*-3HD-*co*-3HDD) (red bar). Mean ± S.D., N = 3 with each N derived from polymer generated from a separate fermentation. One-way ANOVA with Bonferroni's post-hoc test: ∗∗∗∗P < 0.0001. (**C**) The jet lag length attained from the MEW of P(3HO-*co*-3HD) (blue bar) and P(3HO-*co*-3HD-*co*-3HDD) (red bar). Mean ± S.D., N = 3 separate video recordings of MEW each utilising polymer attained from a separate fermentation. Unpaired two-tailed *t*-test: ∗∗P < 0.01. Representative image of: (**D**) a fibrous MEW-PHA scaffold with edge loops (white arrows) and fibre extensions (red arrow). Scale bar represents 0.5 cm. (**E**) Accurate fibre deposition at the edge of the outer box upon modulation of the acceleration and deceleration of the XY stage. Scale bar represents 100 μm. (**F**) A fibrous MEW-PHA scaffold with supporting fibres following detachment from the underlying microscope slide. The outer box (black dotted lines) and supporting y-axis fibres (white arrows) are highlighted. Scale bar represents 0.5 cm. (**G**) Detached porous MEW-PHA scaffold. Scale bar represents 0.5 cm. The anisotropic pores of (**H**) a single-layer porous MEW-PHA scaffold (scaffold A) and (**I**) multi-layer MEW-PHA scaffold (scaffold B). Scale bars represent 100 μm. The (**J**) length, (**K**) width, (**L**) calculated diagonal length, and (**M**) aspect ratio of pores within scaffold A and B. (**J-L**): Mean indicated by ‘+’ sign and whiskers represent minimum and maximum values. (**M**): Mean ± S.D., N = 3 separate scaffolds. Unpaired two-tailed *t*-test revealed no significant difference. (**N**) Scanning electron microscopy image showing the five layers of the MEW-PHA scaffold. (**O**) Topography of the uppermost fibres of scaffold B following post-fabrication cooling. Scale bars represent 50 μm (**P**) Stress-strain curve of scaffold B. (**Q**) Calculated apparent-ultimate tensile strength (σ_u_), -elastic modulus (E), and -elongation at break (ɛ_b_) of scaffold B and P(3HO-*co*-3HD) solvent cast-derived strips. Mean ± S.D., N = 4-6 separate scaffolds or strips fabricated from P(3HO-*co*-3HD) arising from three separate fermentations. (For interpretation of the references to colour in this figure legend, the reader is referred to the Web version of this article.)

MCL-PHAs were attained through the nitrogen-limited batch fermentation of *Pseudomonas mendocina* CH50. ^13^C NMR confirmed the attainment of the homopolymer, Poly(3-hydroxyoctanoate), P(3HO), the copolymer, Poly(3-hydroxyoctanoate-*co*-3-hydroxydecanoate), P(3HO-*co*-3HD), and the terpolymer, Poly(3-hydroxyoctanoate-*co*-3-hydroxydecanoate-*co*-3-hydroxydodecanoate), P(3HO-*co*-3HD-*co*-3HDD), when sodium octanoate, glucose, or coconut oil, were provided as the carbon source, respectively, during fermentation (Fig. 1A, S1A-C). Their low glass transition temperature (T_g_), as ascertained via differential scanning calorimetry (DSC), highlighted the elastomeric nature of these biopolymers (Fig. S1D), which was further confirmed by their high elongation at break (ɛ_b_) (Fig. S1E). Moreover, their relatively low melting temperatures (T_m_) of 47.87 ± 0.55 °C, 52.13 ± 0.46 °C and 57.10 ± 2.03 °C for P(3HO-*co*-3HD-*co*-3HDD), P(3HO-*co*-3HD) and P(3HO), respectively (Fig. S1F), rendered them amenable to fabrication via MEW. However, gel permeation chromatography revealed their relatively high M_w_ of 297.47 ± 14.87 kDa, 281.16 ± 15.42 kDa, and 655.83 ± 19.05 kDa for P(3HO-*co*-3HD-*co*-3HDD), P(3HO-*co*-3HD), and P(3HO), respectively (Fig. 1B), thereby resulting in a disparity between their low T_m_ and the temperature required to establish a stable polymer jet under a constant pressure and voltage (the writing temperature). Indeed, polymer jets could be attained from P(3HO-*co*-3HD) and P(3HO-*co*-3HD-*co*-3HDD) (but not from P(3HO)) only after heating to nearly double their T_m_ (95 °C).

Further, these polymer jets experienced a lag, characterised by an elongated distance between the position directly below the spinneret and the point where the jet was deposited onto the XY stage. The jet lag length attained from the MEW of P(3HO-*co*-3HD-*co*-3HDD) was 1.09 ± 0.03 cm (Fig. 1C), greater than the dimensions of the scaffolds 1 cm^2^ outer box (Fig. S2A), therefore, this terpolymer was not suitable for MEW at this high resolution. On the other hand, this phenomenon was less pronounced with P(3HO-*co*-3HD) (Fig. 1C), although it did perturb the extremities of the emerging MEW-PHA scaffolds (Fig. S2B) which in turn, impinged on the detachment and handleability of the scaffold. Consequently, several refinements were implemented (as described in detail within the Materials and Methods section) to fabricate reproducible and handleable MEW-PHA scaffolds from P(3HO-*co*-3HD).

Indeed, MEW-PHA scaffolds were fabricated by moving the XY stage at 5 mm/s. This relatively slow translation speed ensured that the deposition rate of the polymer jet was correlated with the movement of the XY stage. This is a critical step, as moving the stage too slowly causes excessive fibre deposition per mm, resulting in sinusoidal fibres, whilst moving it too quickly perturbs the accurate deposition of the fibre, again impinging on the reproducibility and overall 3D architecture of the scaffold [52]. Faithful fabrication of the corners of the scaffold was facilitated through the introduction of 0.5 cm^2^ projections emanating from the scaffolds outer box, termed ‘edge loops’ (Fig. 1D–S2A). Further, to ensure precise fabrication of the edges of the fibres that constituted the main body of the scaffold, the deceleration and acceleration rates of the XY stage were modulated between consecutive fibres. Indeed, enhancing the deceleration rate to 1.5 G as the writing of one fibre neared completion, and reducing the acceleration rate to 0.01 G as the subsequent fibre began to be written, proved effective in further mitigating the effects of the jet lag. Conceptually, this ensured the polymer jet was correctly positioned in relation to the XY stage, thus resulting in a more faithful fibre deposition at the edge of the outer box (Fig. 1E). In addition, fibres were also extended 0.5 cm on either side of the outer box to ensure any residual incidences of poor edge formation occurred outside the main body of the scaffold (Fig. 1D).

The non-homogeneous flow of the P(3HO-*co*-3HD)-derived jet also impinged on scaffold reproducibility. This was likely due to the incomplete melting of the loaded bulk polymer that led to the sporadic deposition of large volumes of polymer that perturbed the local environment of the scaffold (Fig. S2C). As the fibre diameter prior to and after this large deposition of polymer was largely unchanged, it was unlikely that this non-homogeneous flow was due to fibre pulsing. Therefore, rather than utilising a single larger piece of polymer, subcentimeter pellets of P(3HO-*co*-3HD) were employed to facilitate homogeneous melting which in turn achieved consistent polymer flow.

Initial fibrous MEW-PHA scaffolds were comprised of fibres in a single (x) axis (Fig. 1D); however, these scaffolds were not readily detachable; therefore, supporting fibres spaced every 250 μm were deposited in the y-axis to enhance scaffold stability during detachment from the underlying microscope slide (Fig. 1F). This concept was further developed through the deposition of these y-axis fibres at smaller, regularly spaced increments of either 150 μm or 200 μm. The resultant MEW-PHA scaffolds displayed superior handleability (Fig. 1G) and, due to the presence of a greater number of fibres relative to the unidirectional fibre containing MEW-PHA scaffolds, offered a larger surface area for cell seeding. Moreover, anisotropic pores could be attained by controlling the distance between neighbouring fibres that, in turn, resulted in structurally anisotropic scaffolds (Fig. 1H and I).

Owing to this anisotropic nature, the achievable resolution and reproducibility of MEW with P(3HO-*co*-3HD) was determined through the calculated diagonal length (CDL) of the pore that considered the length and width of the pore rather than the area. Pores within single-layer porous MEW-PHA scaffolds (scaffold A, Fig. 1H) had an average length of 104.85 ± 6.07 μm (Fig. 1J) and an average width of 140.54 ± 2.43 μm (Fig, 1K) resulting in a CDL of 176.63 ± 2.91 μm (Fig. 1L). This was within 10 % of the intended diagonal length (IDL, 180.28 μm), as specified during computer-aided design (CAD) of the scaffold (Fig. S2D). Therefore, MEW could be conducted with a high M_w_ MCL-PHA at this resolution, and this could be achieved reproducibly with high fidelity, as evidenced by the low interquartile range (IQR) of the CDL (Fig. 1L–S2E).

Although scaffold A could be detached without deformation, it was not readily handleable. Therefore, multi-layer porous MEW-PHA scaffolds were fabricated to retain the structural integrity of the scaffold and improve handleability. A minimum of five layers was required to achieve this. Assessment of pore dimensions within these multi-layer porous MEW-PHA scaffolds revealed a significant reduction in pore size relative to their single-layer variant (Fig. S2F). This reduction was driven, in part, by the flattening and subsequent expansion of the underlying molten layer as the overlaying fibres were deposited, leading to an encroachment of the pore space. The CAD files specifying the precise geometry of the scaffolds were therefore modified to incorporate larger initial pores (100 μm × 200 μm) that could compensate for this expansion. Further, to minimise the perturbations of the underlying layer, 1 min ‘printing pause’ steps were introduced into the MEW protocol. This allowed for the concurrent cooling of the molten fibres and dissipation of electrical charge accumulated during the writing process, which would otherwise be detrimental to inter-scaffold layering. Moreover, upon completion of writing, scaffolds were placed at −20 °C to expedite cooling and solidification of the molten scaffold. Although the pores within the resultant scaffold (scaffold B, Fig. 1I) were smaller than the dimensions specified in the CAD file (Fig. S2D), this modified design successfully compensated for the fibre expansion-mediated perturbations thereby ensuring the attainment of scaffolds with pore dimensions comparable to the single layer scaffold A (Fig. 1J–L). In addition, scaffold B could be fabricated reproducibly resulting in pores with an aspect ratio of 1:1.36 ± 0.16 arbitrary units (A.U.), in line with that of scaffold A (1:1.35 ± 0.12 A U.) (Fig. 1M). The printing pause steps also facilitated the faithful deposition of fibres on one another (Fig. 1N); however, the rapid post-fabrication cooling introduced crater-like topography on the uppermost fibre (Fig. 1O).

Given the highly handleable nature of these scaffolds, their mechanical properties were next ascertained via tensile testing to determine whether they would be suitable for CTE. In comparison to P(3HD-*co*-3HO) strips derived from solvent cast films, the porous architecture of scaffold B resulted in several points of failure (Fig. 1P) and a significantly reduced apparent elongation at break (ɛ_b_, 108.51 ± 24.77 % relative to 410.23 ± 95.63 %, Fig. 1Q). Moreover, a significant reduction in apparent ultimate tensile strength (σ_u_) and apparent elastic modulus (E) was observed relative to the polymer strips (1.08 ± 0.42 MPa and 1.76 ± 0.50 MPa, respectively relative to 10.10 ± 3.58 MPa and 8.84 ± 2.20 MPa, Fig. 1Q). Nevertheless, scaffold B demonstrated high elastomericity and was stiffer than native myocardium, indicating mechanical properties suitable for the development of a cardiac patch.

### 3.2 MEW-PHA scaffolds supported hPSC-cardiovascular cells and induced the structural maturation of hPSC-CMs

**Fig. 2:**
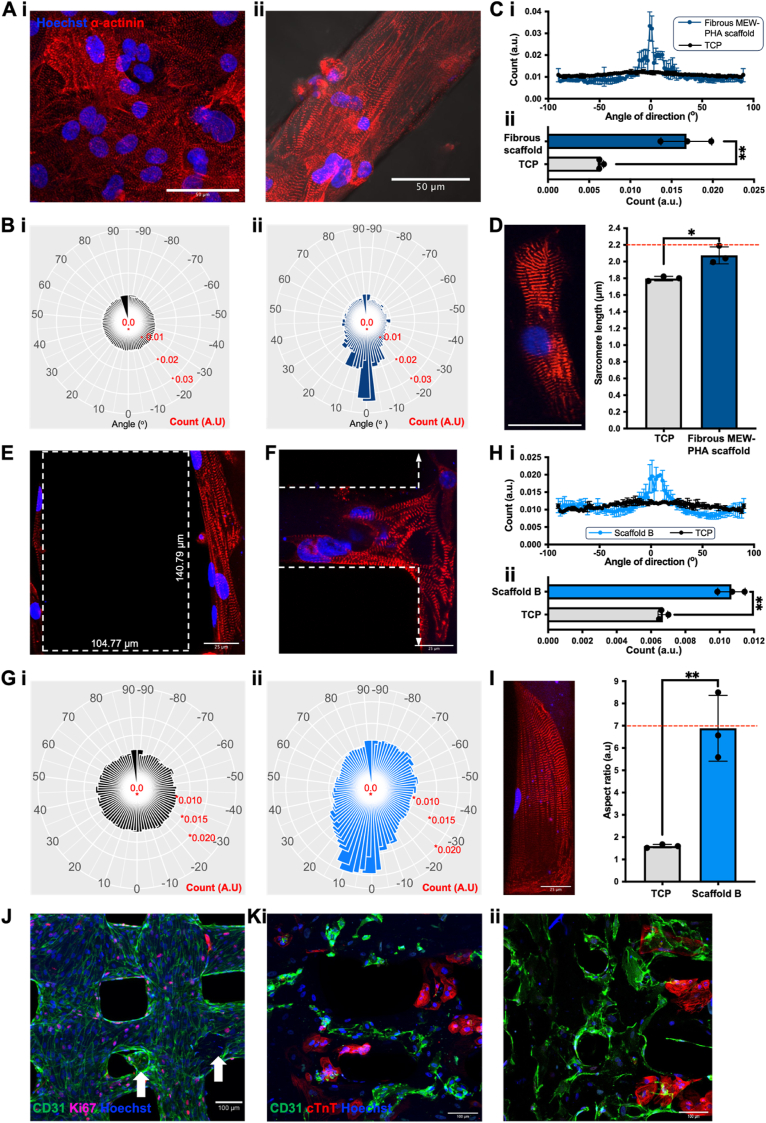
In vitro structural characterisation of hPSC-cardiovascular cell-laden MEW-PHA scaffolds: (**A**) Representative immunofluorescent images of hPSC-CMs seeded on (**i**) tissue culture plastic (TCP) and (**ii**) a fibrous MEW-PHA scaffold. Scale bars represent 50 μm. (**B**) Rose diagram visualisation of hPSC-CM sarcomere alignment following culture on (**i**) TCP or (**ii**) fibrous MEW-PHA scaffolds. (**C**) Quantification of (**i**) sarcomere alignment by way of the (**ii**) count value at half maximum. Mean ± S.D., N = 3. Unpaired two-tailed *t*-test: ∗∗P < 0.01. (**D**) (**i**) Representative immunofluorescent image of a hPSC-CM following one-week of culture on the fibrous MEW-PHA scaffold. Scale bar represents 35 μm. (**ii**) Quantification of the hPSC-CM sarcomere length following culture on either TCP or fibrous MEW-PHA scaffolds. The dotted red line denotes the reported sarcomere length of adult CMs. Mean ± S.D., N = 3. Unpaired two-tailed *t*-test: ∗P < 0.05. (**E-F**) Representative immunofluorescent images of hPSC-CMs seeded on scaffold B, revealing no interaction within the scaffold pore (outlined by the dotted white box), but rather alignment in the direction of the fibre to which they adhered, as demonstrated by the dotted white arrows. Scale bars represent 25 μm. (**G**) Rose diagram visualisation of hPSC-CM sarcomere alignment following culture on (**i**) TCP or (**ii**) scaffold B. (**H**) Quantification of (**i**) sarcomere alignment by way of the (**ii**) count value at half maximum. Mean ± S.D., N = 3. Unpaired two-tailed *t*-test: ∗∗P < 0.01. (**I**) The aspect ratio of hPSC-CMs following this divergent culture. The dotted red line denotes the reported aspect ratio of adult CMs. Mean ± S.D., N = 3. Unpaired two-tailed *t*-test: ∗∗P < 0.01. (**A-I**) hPSC-CMs were cultured for one week on the different culture substrates and were stained for the cardiomyocyte marker α-actinin (red) and the nuclear marker Hoechst (blue). Representative immunofluorescent images of (**J**) hPSC-CMVECs seeded for two weeks on scaffold B and stained for the endothelial marker CD31 (green), the proliferation marker Ki67 (pink), and Hoechst (blue). The white arrows highlight hPSC-CMVECs infiltrating and populating pores. Scale bar represents 100 μm. (**K**) Co-culture of hPSC-CMs and hPSC-CMVECs following (**i**) one- and (**ii**) two-weeks of culture on scaffold B. Cells were stained for the endothelial marker CD31 (green), the cardiomyocyte marker cardiac troponin T (cTnT, red), and Hoechst (blue). Scale bars represent 100 μm. (For interpretation of the references to colour in this figure legend, the reader is referred to the Web version of this article.)

The biocompatibility of the MEW-PHA scaffolds was next evaluated by seeding hPSC-cardiovascular cells onto their surface. In contrast to other polymer studies, where the surface must first be pretreated with fibronectin or collagen [23,24], MEW-PHA scaffolds supported cells without the addition of such extracellular matrix proteins. Indeed, a contractile monolayer of hPSC-CMs was observed on the fibrous MEW-PHA scaffolds within one week of seeding (Fig. 2A), by which point hPSC-CMs had aligned in the direction of the fibre, as determined by a less variable sarcomere organisation relative to fibronectin-coated tissue culture plastic (TCP) (Fig. 2B and C). This was also associated with an increase in sarcomere length relative to TCP, to levels in line with adult CMs (∼2.2 μm) [53] (Fig. 2D).

Although the porous MEW-PHA scaffolds comprised fibres in both axes, hPSC-CMs cultured upon scaffold B continued to adhere to the fibres of the scaffold rather than interacting with the pore (Fig. 2E) and aligned in the direction of the particular fibre on which they adhered (Fig. 2F), once more resulting in a less variable sarcomere organisation (Fig. 2G and H). Further, the hPSC-CMs demonstrated an elongated and anisotropic morphology resulting in an aspect ratio comparable to mature, adult CMs in vivo (∼7:1) [54] (Fig. 2I). Neither the medium-term culture of hPSC-CMs on TCP (Fig. S3) nor their culture upon P(3HO-*co*-3HD)-derived solvent cast films (Fig. S4) induced structural alignment or increased sarcomere size, thus confirming that the structural maturation observed upon the MEW-PHA scaffolds was driven by the fibrous scaffold architecture generated during the MEW process.

The ability of scaffold B to support hPSC-CMVECs was also evaluated. hPSC-CMVECs adhere to collagen IV-coated TCP that supports their viability and proliferation [27]. However, in the absence of collagen IV pretreatment, hPSC-CMVECs adhered to the fibres of the scaffold and proliferated to form a confluent monolayer following two weeks of in vitro culture (Fig. 2J). This proliferative capacity, as evidenced by expression of the proliferative marker Ki67, was sustained upon the attainment of confluency, thus resulting in the infiltration of hPSC-CMVECs into the scaffold pores, where they presumably adhered to the sides of the underlying fibres of the scaffold (Fig. 2J). Subsequently, a co-culture of hPSC-CMs and hPSC-CMVECs was established at an initial 3:1 ratio, which, following two weeks of in vitro culture and robust hPSC-CMVEC proliferation, resulted in an approximate 1:1 ratio of hPSC-CMs: hPSC-CMVECs (Fig. 2K), in line with the observation that each endogenous CM is surrounded by a dense network of cECs [24]. As scaffold B efficiently maintained both hPSC-cardiovascular cell types, facilitated the alignment and structural maturation of hPSC-CMs, and was readily handleable, further evaluation and development of the cardiac patch was conducted with this scaffold variant.

### 3.3 hPSC-CMs demonstrated functional improvement on the MEW-PHA scaffolds

**Fig. 3:**
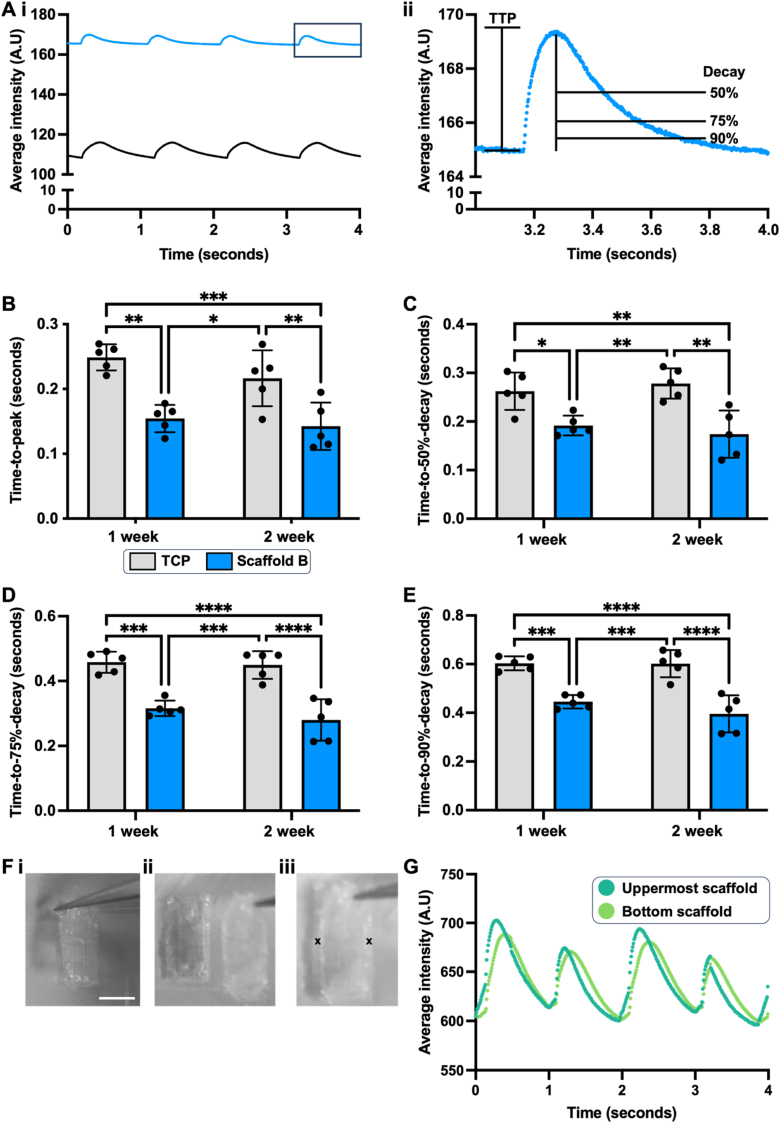
In vitro functional assessment of hPSC-CM-laden MEW-PHA scaffolds: (**A**) Representative intensity time traces attained from the optical mapping of Fluo-4-AM-loaded hPSC-CMs that had been cultured on (**i**) TCP (black trace) or scaffold B (blue trace) for one week. (**ii**) Inset of the trace attained from scaffold B with time-to-peak (TTP) and time-to- 50 %-, −75 %-, and 90 %-decay highlighted. Calcium (Ca^2+^) handling kinetics of hPSC-CMs cultured on TCP (grey bars) or scaffold B (blue bars) for 1 or 2 weeks: (**B**) TTP Ca^2+^ transient, time-to- (**C**) 50 %-, (**D**) 75 %-, and (**E**) 90 %-decay of Ca^2+^ transient. Mean ± S.D., N = 5. Two-way ANOVA with Tukey's post-hoc test: ∗P < 0.05, ∗∗P < 0.01, ∗∗∗P < 0.001, ∗∗∗∗P < 0.0001. (**F**) Generation of the MEW-PHA cardiac patch: (**i**) single scaffolds are folded in half lengthwise to attain 0.5 cm wide scaffolds, (**ii**) two of these are placed on top of each another and (**iii**) held in place with two sutures as denoted by the black crosses. Scale bar represents 0.5 cm. (**G**) Representative intensity time trace from the uppermost (dark green) and bottom scaffold (light green) within a cardiac patch following point stimulation of the former. (For interpretation of the references to colour in this figure legend, the reader is referred to the Web version of this article.)

Optical mapping studies were performed to investigate the calcium (Ca^2+^) handling kinetics of the hPSC-CMs seeded on scaffold B. The time taken for Ca^2+^ to be released from the sarcoplasmic reticulum (the time-to-peak Ca^2+^ transient, TTP) and the time taken for its reuptake into the various Ca^2+^ stores (time-to-50 %-, −75 %-, and −90 %-decay of the Ca^2+^ transient) were therefore determined (Fig. 3A). A single week of culture on scaffold B resulted in a significant enhancement of all four parameters relative to TCP controls (Fig. 3B–E), indicative of functional improvement of the hPSC-CMs. Although sustained, this was not further enhanced by an additional week of culture.

The effect of the hPSC-CMVECs on the Ca^2+^ handling kinetics of the hPSC-CMs was also examined through comparison of these parameters attained from monocultures and co-cultures. To account for the effects of the exogenous VEGF-A present in the hPSC-CMVEC maintenance media, both sets of cultures were maintained in VEGF-A-containing co-culture media. In contrast to scaffold B monocultures that were maintained in hPSC-CM media (Fig. 3B), the TTP of hPSC-CM monocultures seeded on scaffold B for either one or two weeks of culture was no longer significantly enhanced relative to their TCP controls when maintained in this VEGF-A-containing media (Fig. S5A). However, improvements in decay kinetics remained largely similar, although the enhancement in time-to-75 %- and −90 %-decay, previously observed between monoculture TCP and monoculture scaffold B at one week (Fig. 3D and E) was now only attained following two weeks of monoculture on scaffold B when maintained in the co-culture media (Fig. S5C and D). Interestingly, co-culture on scaffold B could still achieve this enhancement relative to the co-culture TCP at one week, suggesting that in the absence of the hPSC-CMVECs, the co-culture media and the presence of exogenous VEGF-A delayed the enhancement of these decay kinetics in hPSC-CM monocultures seeded on scaffold B.

### 3.4 A synchronously contracting MEW-PHA cardiac patch was attained through the stacking of scaffolds

As an additional week of culture on scaffold B did not further enhance the structural maturation nor functional improvement of hPSC-CMs, the final cardiac patch was constructed by maintaining the cell-laden scaffolds independently for one week and thereafter stacking them upon one another to attain the final cardiac patch. Owing to the dimensions of the murine LV, the 1 cm^2^ scaffolds were firstly folded in half with the cell-seeded sides facing outward prior to stacking, resulting in a patch composed of two identical scaffolds in which cells seeded on either scaffold were in direct contact with each other (Fig. 3F–S6). The resultant patch was cultured for a further week in vitro to attain electrical coupling between the two scaffolds, as evidenced by the synchronous contraction of hPSC-CMs on the bottom scaffold following point stimulation of the uppermost scaffold (Fig. 3G).

### 3.5 In vivo functional analysis of the different MEW-PHA cardiac patches

**Fig. 4:**
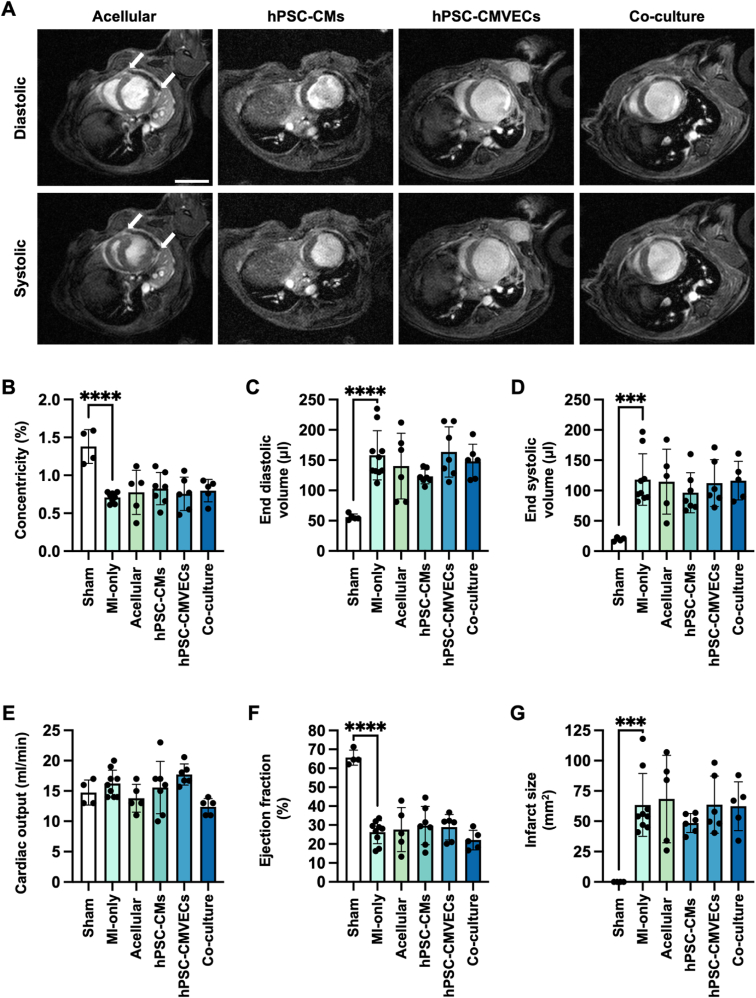
In vivo assessment of the MEW-PHA cardiac patches: (**A**) Representative cardiac cine MRI images of the infarcted murine heart, 4 weeks after concurrent MI and application of either an acellular, hPSC-CM monoculture, hPSC-CMVEC monoculture, or co-culture patch. Images were acquired in the short axis during diastole and systole. The white arrows mark the edges of the patch. Scale bar represents 1 mm. Quantification of the (**B**) concentricity, (**C**) end diastolic volume, (**D**) end systolic volume, (**E**) cardiac output, (**F**) ejection fraction, and (**G**) infarct size of the murine heart across different treatment groups at this time point. Mean ± S.D., N = 4–9. One-way ANOVA with Bonferroni's post-hoc test: ∗∗∗P < 0.001, ∗∗∗∗P < 0.0001.

To investigate the effect of the different cardiac patches on both post-MI cardiac structure and function, cardiac cine MRI was conducted four-weeks post-MI and concurrent patch administration (Fig. 4A). Three-lead ECG measurements were used to determine the induction of STEMI within 1 min of permanent LAD ligation and thus inclusion of these animals in the study (Fig. S7). MEW-PHA patches contracted and relaxed in synchrony with the LV, illustrating the elastomericity of the P(3HO-*co*-3HD)-derived MEW-PHA scaffolds and indicating high compliance with the cyclic nature of the contracting myocardium. However, the patches were unable to ameliorate the eccentric remodelling of the LV as determined by reduced LV concentricity post-MI (Fig. 4B). In turn, this was associated with an increase in both the end diastolic- and systolic-volumes of the LV relative to sham operated mice (Fig. 4C and D). Given the simultaneous increase in both of these LV volume parameters, stroke volume was not significantly impinged upon post-MI and thus cardiac output (CO) remained consistent across all treatment groups relative to MI-only animals (Fig. 4E). In addition, this concurrent administration of any of the cardiac patches at the point of infarction was unable to prevent post-MI functional decline, as determined by a reduction in LVEF (Fig. 4F). Late gadolinium enhancement (LGE)-MRI also confirmed the formation of substantial scar tissue, which was comparable in size across all treatment groups relative to MI-only animals (Fig. 4G).

### 3.6 MEW-PHA cardiac patches were well tolerated in vivo

**Fig. 5:**
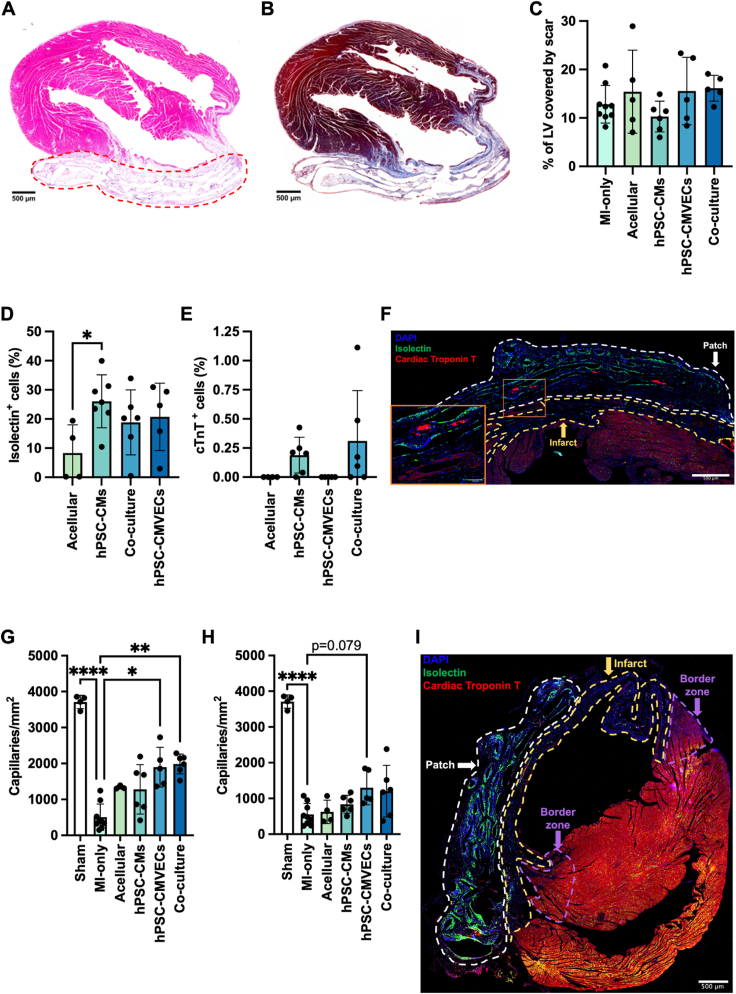
Histological examination of the MEW-PHA cardiac patches: Representative images of murine hearts excised 4 weeks after concurrent MI and patch application, stained for (**A**) Haematoxylin and eosin and (**B**) Masson's Trichrome staining. The patch is highlighted by the dotted red line. Scale bars represent 500 μm. (**C**) The percentage of the LV that is comprised of a fibrotic scar. Mean ± S.D., N = 4–9. One-way ANOVA with Bonferroni's post-hoc test revealed no significant difference between groups. The percentage of (**D**) isolectin and (**E**) cardiac troponin T (cTnT) positive cells within the different patches at the 4-week experimental end point. Mean ± S.D., N = 4–9. One-way ANOVA with Bonferroni's post-hoc test: ∗P < 0.05. (**F**) Representative immunofluorescent image of the patch (dotted white line) attached to the infarcted LV (infarct, dotted yellow line) at 4 weeks, containing isolectin-positive cells (green) and hPSC-CMs, stained for cTnT (red), that have maintained their structurally mature phenotype (inset). Scale bar represents 500 μm. Quantification of isolectin-positive capillaries per mm2 of the (**G**) infarct and (**H**) the border zone following application of the different patches. Mean ± S.D., N = 4–9. (**G**) Kruskal-Wallis test with Dunn's post-hoc test. (**H**) Brown-Forsythe test with Welch ANOVA and Dunnett's T3 post-hoc test. (**G-H**) ∗∗P < 0.01, ∗∗∗P < 0.001, ∗∗∗∗P < 0.0001. (**I**) Representative immunofluorescent image of the murine myocardium 4 weeks after administration of the co-culture patch. The infarct (dotted yellow line), infarct border zone (dotted purple line), and patch (dotted white line) are delineated. Scale bar represents 500 μm. (For interpretation of the references to colour in this figure legend, the reader is referred to the Web version of this article.)

Subsequent histological examination of murine hearts excised following cardiac cine MRI further substantiated the excellent biocompatibility of the P(3HO-*co*-3HD)-derived cardiac patch that displayed minimal fibrosis or capsule formation, suggesting the absence of a FBR (Fig. 5A and B). This was further supported by the clear visualisation of the patch following explanation of the heart (Fig. S8 A, C) and no evidence of a thick capsule following administration of a single acellular MEW-PHA scaffold to the LV of a healthy rat for one week as part of a pilot experiment (Fig. S8B). Rather, the patch remained well adhered to the LV. Moreover, the patch architecture and inter-patch layers, created by the folding and stacking of the two MEW-PHA scaffolds (Fig. 3F–S6), were clearly visible suggesting that extensive bioresorption of the scaffold had not yet taken place (Fig. 5A). Masson's Trichrome staining corroborated the LGE-MRI observations (Fig. 4G), highlighting a large fibrotic scar that was not resolved following the application of any of the cardiac patches (Fig. 5B and C).

Although NSG mice lack functional T and B cells, they retain components of the innate immune system, including macrophages. Whilst these are functionally perturbed, thus limiting their capacity to clear the exogenously delivered hPSC-cardiovascular cells [55], they remain capable of infiltrating tissues and therefore provide insight into the innate immune response and the FBR to the MEW-PHA patches. As such, DAB staining for the macrophage marker Iba1 was conducted revealing minimal macrophage infiltration within the acellular patches, once again highlighting the biocompatibility of the P(3HO-*co*-3HD)-derived cardiac patch (Fig. S8A and B). In contrast, a significant increase in Iba1-positive cells was noted within hPSC-CM monoculture patches. Whilst this was relatively low, this increase was intriguingly not observed in the co-culture patch, despite also containing hPSC-CMs (Fig. S8D and E). Iba1 expression within the proximal healthy myocardium of treated groups was unchanged relative to sham operated animals (Fig. S8F), suggesting this low-level macrophage infiltration likely arose from circulating macrophages rather than tissue-resident macrophages.

### 3.7 hPSC-CMVEC-containing MEW-PHA patches instigated vascular regeneration within the infarcted myocardium

Immunofluorescent assessment of the patches (Fig. 5D–I) revealed that, although initially devoid of exogenous hPSC-CMVECs, both the acellular and hPSC-CM-only patches exhibited comparable numbers of isolectin-positive cells, indicative of ECs, when compared to patches seeded with hPSC-CMVECs only (Fig. 5D). Interestingly, analysis of the infarcted myocardium revealed that administration of patches seeded with hPSC-CMVECs initiated partial vascular regeneration within the infarct, as determined by increased capillary density within the infarcted myocardium relative to MI-only animals (Fig. 5G–I). Of note, no statistically significant difference in capillary density within the border zone was identified between the different patch groups (Fig. 5H), potentially indicating that the capillaries detected within the infarct originated from hPSC-CMVECs migrating from the patch into the infarct, rather than transversing from the healthy myocardium into the infarct via the border zone (Fig. 5I).

However, neither the apparent reactive angiogenic response mounted by the host, nor vascularisation of the infarct facilitated the longer-term retention of the majority of hPSC-CMs within the patch (Fig. 5E). Rather, only a small percentage of cells within the hPSC-CM monoculture patch (0.19 ± 0.15 %) and co-culture patch (0.31 ± 0.43 %) stained positive for the CM marker cTnT. The retained hPSC-CMs conserved the mature, anisotropic morphology that developed during early in vitro culture (Fig. 5F).

## 4 Discussion

In this study, we describe the optimisation of MEW with the novel sustainable biopolymer, high M_w_ P(3HO-*co*-3HD), to attain reproducible, structurally anisotropic, and handleable MEW-PHA scaffolds that were capable of sustaining hPSC-cardiovascular cells. These scaffolds induced prompt structural maturation of hPSC-CMs in vitro alongside improvements in their functional parameters. When incorporated into a cardiac patch, the scaffolds were well tolerated in vivo and hPSC-CMVEC-containing patches increased vascularisation within the infarcted myocardium.

The biocompatibility of MCL-PHAs, alongside their mechanical properties, makes them ideal candidates for CTE. Further, the potential to attain reproducible MCL-PHA scaffolds at scale without cytotoxic solvents renders MEW an attractive fabrication technique for CTE solutions intended for future clinical translation. However, polymer jets attained via MEW have a viscosity an order of magnitude greater than if they were derived via solution electrospinning [56]. As such, MEW scaffolds intended for use in cardiac patches have predominantly been produced from either low M_w_ PCL (M_w_ 45 kDa), resulting in the attainment of auxetic MEW-PCL scaffolds that complement the mechanical anisotropy of the myocardium [57], or from PCL co-polymers, including poly-hydroxymethylglycolide-*co*-ε-caprolactone (pHMGCL) (M_w_ 39 kDa), that enhance the alignment of cardiac progenitor cells relative to PCL-derived scaffolds [52]. Overall, all known successful attempts of MEW have used polymers with molecular weights ranging between M_w_ 39 to <200 kDa. Indeed, recent developments in MEW have achieved successful fabrication using 130 kDa PLLA, a notable increase from the conventional yet brittle 45 kDa PLLA utilised for MEW [58]. In contrast, our study presents a significant advancement, allowing for fabrication of intricate, high resolution, reproducible MEW scaffolds with a molecular weight of 281.16 ± 15.41 kDa, twice that of the most advanced protocols for PLLA [58]. Thus, to the best of our knowledge, this study describes the first ever successful MEW of either a high molecular weight polymer or that of any known MCL-PHA. This has the potential to revolutionise the use of MEW for a wide range of high molecular weight polymers leading to the development of a well-defined and bespoke MEW-derived scaffolds for complex tissue engineering applications beyond CTE, and therefore represents a significant advance in additive manufacturing.

In a CTE context, certain MEW-PCL scaffolds require the submersion of scaffolds in rat-derived collagen hydrogels in order to achieve enhanced sarcomere length and contractility of the seeded hPSC-CMs [59]. The use of animal-derived biopolymers, particularly collagen, presents challenges pertaining to its availability, sustainability, and clinical translation potential given these animal-derived biopolymers could facilitate pathogen transmission. Further, xenogeneic collagen may also cause an immune response [60]. The adherence and proliferation of the hPSC-CMVECs, and the structural maturation and alignment of hPSC-CMs outlined within our study required no such pretreatment. Indeed, maturation occurred rapidly within a week of culture, directly on the untreated surface of the P(3HO-*co*-3HD)-derived MEW-PHA scaffolds illustrating the high biocompatibility of this MCL-PHA.

To attain a highly handleable scaffold, each MEW-PHA scaffold consisted of five layers, with the fibres in each layer deposited along both axes. This created small (<200 μm), anisotropic pores within the scaffold that conferred structural anisotropy, capable of maturing and aligning the hPSC-CMs, without causing excessive cell loss through the pores during cell seeding. However, the combination of a relatively high M_w_ polymer and small pores presented several challenges that required optimisation of the interconnected MEW parameters. Indeed, we employed a writing temperature significantly greater than the melting temperature in order to attain polymer jets from the copolymer, P(3HO-*co*-3HD), and the terpolymer, P(3HO-*co*-3HD-*co*-3HDD). Intriguingly, despite its low M_w_, PCL also exhibits a dichotomy between its writing temperature (94 °C) and its T_m_ (54.4 °C) [52]. The emerging MCL-PHA jets, however, exhibited a lag. This was particularly pronounced with P(3HO-*co*-3HD-*co*-3HDD) that continues to crystallise for several weeks after production via fermentation [15], potentially explaining the difference between the jet lag of the two MCL-PHAs despite their similar M_w_. The shortest achievable lag for jets from either MCL-PHA was attained by writing at slow translation speeds and maintaining a short distance between the spinneret and the XY stage. Nevertheless, modulation of the acceleration and deceleration of the XY stage at the fibre edge was required to attain edges more closely representative of the 90° edge stipulated in the CAD file. Additionally, edge loops and extended fibres were employed as redundancy measures to ensure any sporadic emergence of jet lag did not perturb the main body of the scaffold. Traditionally, a quick translation speed and a large distance between the spinneret and the XY stage are utilised to promote fibre thinning [61] resulting in thin nanofibres. However, owing to the high M_w_ of P(3HO-*co*-3HD) and the high resolution sought in this study, a slow translation speed at the minimal distance from the XY stage was required; thus, these parameters could not be exploited to generate submicron MCL-PHA fibres. Further, the effect of voltage on the angle of the jet is particularly pronounced at these slow translation speeds [62], therefore a high voltage was engaged to further minimise lag. Recently, using 45 kDa PCL, Lu et al. [63] demonstrated that high voltage becomes detrimental when fabricating pores smaller than 200 μm. This leads to an offset of subsequent layers in relation to those underneath, thereby disrupting the reproducibility of pores throughout the scaffold. Here, we employed printing pause steps that simultaneously prevented the accumulation of charge within the developing scaffold, thereby enabling efficient inter-scaffold layering, and facilitated the cooling of the thicker fibres, which likely took longer to dissipate heat as compared to thin (∼10 μm) MEW-PCL fibres [52,61]. Further optimisation of the MEW of MCL-PHAs could be undertaken through the assessment of fibre deposition upon a cooled XY stage that expedites cooling of the fibre, thereby potentially preventing it from expanding into the pore space. However, a gradient in temperature between the spinneret and XY stage would influence jet dynamics. The advent of high throughput ‘printomics’, which combines MEW with live microscopy, allows for visual assessment of the jet and the emerging scaffold in real-time [62]. This could be employed to quantify fibre expansion in our current protocol and evaluate the effect of a temperature gradient and cooling of the fibre immediately following its deposition onto the XY stage.

The MEW-PHA scaffolds offer superior maturation of hPSC-CMs relative to other MCL-PHA-based scaffolds. Indeed, solvent cast films are a simple yet handleable cellular scaffold solution and although MCL-PHAs are hydrophobic, P(3HO-*co*-3HD)-derived films evaluated within efficiently supported the culture of hPSC-CMs, forgoing the need for prior pretreatment with an ECM protein. However, beyond this, these 2D films were incapable of enhancing hPSC-CM alignment or maturation. Conversely, fibrous 3D scaffolds are well known for their capacity to align hPSC-CMs. Indeed, electrospun fibres of P(3HO), or its blend with poly(3-hydroxynonanoate-*co*-3-hydroxyheptanoate) P(3HN-*co*-3HHP), facilitate the alignment of seeded hPSC-CMs following two weeks of culture [64]; however, in contrast to the MEW-PHA scaffolds, these scaffolds are not readily handleable nor reproducible. Further, the MEW-PHA scaffolds achieved quicker hPSC-CM alignment, structural maturation, and improvements across all parameters of the calcium transient within one week of in vitro monoculture. Longer in vitro culture did not yield additional enhancements of these parameters, suggesting that a maturation plateau had been reached for these parameters. It is possible that other parameters, for example, isoform switching of known CM maturation-associated genes, occurred during this period; however, this was not assessed in this study. The MEW-PHA scaffolds appeared to drive maturation through their fibrous architecture, with potential contribution from topographical cues on the fibres surface that may have been introduced during the MEW process. As this represents a single biophysical stimulus, it is plausible that its capacity to induce further or complete maturation of hPSC-CMs in vitro is inherently limited. Consequently, a more holistic approach utilising, for example, metabolic maturation media, known to independently enhance Ca^2+^ handling kinetics [65], could provide unique maturation cues to further drive in vitro maturation upon the MEW-PHA scaffolds. Omic technologies including single-cell RNA sequencing (scRNA-seq) should also be employed to concurrently ascertain the molecular mechanisms instigated in response to these different maturation cues, whilst also evaluating the isoform switching of CM genes, as well as identifying novel mediators of hPSC-CM maturation. Recently, an advanced differentiation protocol has generated LV-like hPSC-CMs that display an inherent structural and functional maturity relative to the hPSC-CMs emerging from the differentiation protocol utilised in this study [66]. Intriguingly, EHTs composed of these more mature hPSC-CMs exhibit even greater structural alignment and apparent force generation when compared to EHTs derived from conventional hPSC-CMs. Therefore, it would be pertinent to also evaluate whether the observed maturation plateau is surmountable if the initially seeded hPSC-CMs display an inherent degree of maturity. Nevertheless, the 3D MEW-PHA scaffolds described in our study quickly yielded highly mature hPSC-CMs, complete with enhanced Ca^2+^ handling kinetics, that would be suitable for in vitro drug potency assays and cardiotoxicity screening applications. Indeed, given that the Food and Drug Administration (FDA) now allow for the assessment of new medicines in well-characterised 3D in vitro models [67], intricate biomaterial scaffolds derived from high M_w_ biopolymers fabricated via MEW, which provide the necessary cues to enhance the maturation of the seeded hPSC-derived cells or organoids [68], offer an exciting avenue for the high throughput assessment of new medicines on more-mature hPSC-CMs in vitro.

From a CTE perspective, the concurrent administration of a cardiac patch to the infarcted LV during post-MI coronary artery bypass grafting (CABG) represents an exciting potential therapeutic avenue that aims to quickly restore CMs, and thus contractility, to the infarcted myocardium. Conceptually, this restoration in contractility alongside the mechanical support from the patch that should attenuate eccentric remodelling, could prevent post-MI functional decline towards HFrEF, thereby negating the need to reverse the established end-stage disease. However, following in vivo assessment of the first iteration of MEW-PHA cardiac patches, further optimisation is required to achieve this. Indeed, our rationale was that hPSC-CMVECs would promote rapid vascularisation of the patch, thereby enhancing the viability, retention, and integration of hPSC-CMs within the infarcted LV following synchronised bioresorption of the scaffolds. The ability of cECs to retain CMs is supported by the observation that following MI, the viability of the small number of endogenous CMs that are retained within the infarcted LV is dependent upon whether they are in contact with a microvessel [69]. Whilst patches containing hPSC-CMVECs promoted vascular regeneration within the infarct, they did not improve hPSC-CM retention nor attenuate post-MI functional decline. This indicates that restoring contractile tissue may take precedence over restoring the myocardial vasculature. As the NSG mice is the leading in vivo model of immunodeficiency [70] and has been utilised extensively in the field [71,72], it is unlikely that the exogenous hPSC-cardiovascular cells were removed from the patch via the hosts immune response. Rather, it is plausible that, in the absence of functional vascularisation of the patch, the relatively large number of highly metabolically active exogenous hPSC-CMs quickly succumbed to the hypoxic post-MI environment before sufficient vascularisation could be established.

As the MEW-PHA scaffolds were not embedded within a hydrogel during in vitro culture, the hPSC-CMVECs proliferated and formed a monolayer on the scaffold surface during two-weeks of in vitro culture rather than generating pre-formed vessels. Previous reports outlining the administration of EC-derived cell sheets discuss a rapid and robust angiogenic response resulting in the administered monolayer quickly adopting a tubular morphology in vivo [73]. Vascular regeneration of the infarct was observed, and it is plausible that this was mediated by the hPSC-CMVECs themselves. To this end, the microvasculature is the major site of capillary angiogenesis, a process dependent upon highly migratory tip cells, the markers of which are highly expressed by the hPSC-CMVECs [27]. Thus, these cells may have migrated from the patch into the infarct. This is further supported by no significant increase in capillary density in the border zone of these mice relative to MI-only, suggesting these capillaries did not emerge from the healthy myocardium proximal to the infarct. However, the paracrine effect of the hPSC-CMVECs cannot be discounted. Recently, the isolation and concentrated delivery of exosomes arising from hPSC-ECs improves LVEF [74] indicating that angiocrine factors may be of greater importance than the restoration of blood flow during this initial stage of regeneration. Patches originally devoid of hPSC-CMVECs also expressed isolectin-positive cells at the four-week endpoint suggesting a host-mediated vascular response to both the biomaterial and the hPSC-CMs or their angiocrine factors, a phenomenon routinely observed with CTE solutions [23].

Multi-modal labelling and imaging technologies [75] could be utilised to understand the temporal relationship between patch administration, the clearance of exogenous cells from the patch, and the timing and origin of vascular regeneration of the infarct. This insight could inform modifications to the patch design or composition to better synchronise the achieved vascular regeneration with hPSC-CM delivery, thereby enhancing hPSC-CM retention and cardiac regeneration, which in turn may attenuate post-MI functional decline.

Indeed, if multi-modal tracking studies indicate that hPSC-CMs undergo necrosis shortly after administration, the next generation of MEW-PHA cardiac patches could augment the role of the hPSC-CMVECs. To this end, the concomitant delivery of microvessels with hPSC-CMs has recently been shown to enhance hPSC-CM retention in a rat model of MI [76]. Therefore, hPSC-CMVECs could be incorporated as pre-formed microvessels, by potentially encapsulating the patch within a hydrogel, rather than a confluent monolayer. This approach may enhance hPSC-CM retention by firstly keeping hPSC-CMVECs (now as vessels) within the patch, where they can anastomose with the infiltrating host vasculature to sustain hPSC-CMs rather than infiltrating into the infarcted myocardium to undertake vascular regeneration which, at this stage of the regenerative cascade, does not appear to yield contractile benefits. Secondary to this, the patches could also be derived from P(3HO-*co*-3HD) blended with a hydrophilic biopolymer that promotes and enhances bioresorption of the scaffold. In conjunction, this would allow the potentially functionally vascularised and retained hPSC-CMs to integrate into the infarcted myocardium where they can contribute to LV contractility and thus attenuate post-MI functional decline.

An alternative strategy to improve hPSC-CM retention could instead take advantage of this slow bioresorption. Our observation that the scaffolds macrostructure remained largely intact four-weeks post-administration is in line with sutures and meshes derived from the short-chain length PHA, Poly-4-Hydroxybutyrate (P4HB), in which bioresorption is initiated at a molecular level [77], yet these medical devices are still detectable up to 18 months post-administration [78]. Immature hPSC-CMs are more resilient to hypoxia [79] and could therefore be delivered upon the scaffolds in place of the phenotypically adult-like hPSC-CMs employed within this study. Conceptually, their superior resilience, combined with the scaffold's retained fibrous architecture that drives hPSC-CM maturation, could allow for greater retention during the period in which the hPSC-CMVEC-derived microvessels are anastomosing with the host vasculature. Thereafter, these immature hPSC-CMs could undergo in situ maturation upon the scaffold. Although immature hPSC-CMs may pose a greater arrhythmogenic risk, as observed following their injection into the myocardium [80], this abates as the hPSC-CMs mature and integrate into the myocardium. Further, the slow bioresorption of the cardiac patch could also offer electrophysiological benefits by physically separating the immature, pro-arrhythmogenic hPSC-CMs from direct contact with the myocardium, thereby diminishing this arrhythmogenic risk.

Beyond the limited retention of hPSC-CMs, the inability of the MEW-PHA scaffolds to prevent post-MI eccentric remodelling was another major factor underlying the inability of the patch to attenuate post-MI functional decline. This was despite the MEW-PHA scaffolds being significantly stiffer than the healthy myocardium, which, in theory, was supposed to constrain the infarcted LV. More recently, the internal geometry of a patch appears to be more important than its elastic modulus in preventing this remodelling process. Indeed, a synthetic gelatin methacrylate: polyethylene glycol diacrylate (GelMA:PEGDA) hydrogel, which has a significantly lower elastic modulus (∼50–130 kPa) than the MEW-PHA scaffolds, attenuated remodelling. This was attributed to the patches ‘fourth dimension’ that recaptured the helical nature of the myofibres [81]. As the construction of the MEW-PHA cardiac patch in our study involved the stacking two scaffolds, it is conceivable that the successive MEW-PHA scaffold could be stacked at an angle rather than directly above. This may produce a helical-like configuration that could potentially attenuate eccentric remodelling and partially prevent post-MI functional decline.

Preliminary histological analysis from the ongoing BioVAT-HF-DZHK20 Phase 1/2 clinical trial, in which EHMs are administered to end stage HFrEF patients, has resulted in the number of administered EHMs being doubled from 10 to 20 EHMs [82]. In turn, 800 × 10^6^ hPSC-CMs and stromal cells will now be delivered to the infarcted human LV which is approximately the total number of CMs lost from within the adult human myocardium following MI. In our study, the number of hPSC-CMs incorporated onto the scaffold was based on the number of cells required to achieve a confluent monolayer, whereas the total number of hPSC-CMs delivered to the infarcted murine myocardium was dependent upon how many scaffolds were incorporated into the cardiac patch, and this was determined by scaling relative to other pre-clinical models. Indeed, whilst the NSG mouse model has become a mainstay in the investigation of exogenous cells, studies have often focused on the use of hPSC-derived cardiac progenitor cells, which expand and terminally differentiate following injection into infarcted LV [71,72]. Although hPSC-CM-containing patches have also been evaluated in the NSG mouse, the total number of cells administered is not specified [81]. Therefore, the number of hPSC-CMs delivered to the infarcted mouse LV in our study was guided by comparing the weight of the mouse heart (0.15 g) to that of the hearts of other pre-clinical models [83]. A 2.5- to 5-fold greater number of hPSC-CMs/g of heart weight was delivered via the MEW-PHA cardiac patches compared with the number delivered via the EHMs/EHTs to infarcted monkey and rabbit hearts, respectively [23,82]. Somewhat counterintuitively, however, this greater number of cells may be detrimental to their retention in the absence of rapid functional vascularisation. Indeed, the high metabolic demands of hPSC-CMs means that, without sufficient vascularisation and influx of nutrients, the large number of exogenous cells could further exacerbate the hypoxic environment following MI resulting in necrosis of the delivered cells. The extent of this may be reduced in previous studies that deliver a relatively lower number of exogenous hPSC-CMs from which a fraction remain viable until the host vasculature infiltrates the patch.

Whilst a greater relative number of hPSC-CMs were delivered in our study, this takes place on a scaffold that is relatively large for the murine heart. As such, these cells are distributed across a larger area resulting in a cell density that is below that utilised in previous studies [84]. Therefore, even if cell retention within the current patch iteration were improved, the resulting graft may be too thin and fail to penetrate deeply into the myocardium, potentially limiting its ability to enhance contractility. As such, in addition to the above modifications that will be conducted in the next iteration of MEW-PHA cardiac patches, cell density will be increased either by delivering a greater number of hPSC-CMs, for example, by incorporating a greater number of scaffolds per patch, or by utilising smaller patches in which both sides of the patch are seeded. This will also require the rapid establishment of a functional vasculature.

Finally, the permanent LAD ligation model utilised in this study offers robust experimental utility by reproducibly creating large transmural infarcts that reduce LVEF and induce LV remodelling as evidenced in our study. Further, this model is also associated with inversion of the force-frequency relationship, desensitisation of β-adrenergic receptors, and residual transmural islands of CMs [23]. As such, it is deemed a suitable model for studying ischemic injury and regeneration, particularly in mice, which are more resilient than humans and require a larger infarct to significantly reduce LVEF and induce remodelling [38]. However, utilisation of this model is not only limited to mice with large animal models investigating EHTs also utilising permanent LAD ligation [23]. In the clinic, PCI is intended to reperfuse the occluded coronary artery in a timely manner, restoring blood flow to the infarcted myocardium and reducing ischemic injury to the myocardium albeit with the risk of reperfusion injury. However, up to 40 % of patients with STEMI present 12 h after onset of symptoms rendering PCI less effective [85]. Further, patients may either be ineligible for PCI or may undergo this procedure in a timely manner yet demonstrate a lower efficacy of revascularisation owing to comorbidities [86]. As such, these patients experience a long-term ischaemic condition that is modelled by the permanent LAD ligation model utilised in this study. On the other hand, those who undergo effective, timely PCI (and thus experience reperfusion injury) may be better modelled by the ischemia reperfusion injury (IRI) model of MI; however, caution must be taken in drawing parallels to the human condition. To this end, the failure of therapies to prevent reperfusion injury in clinical trials despite promising preclinical studies has been attributed to the lack of experimental model that closely recapture the myocardial injury seen from reperfusion injury in humans [87]. Moreover, whilst IRI has been utilised in the evaluation of cardiac patches in large animal models [82] and can also be refined to generate large infarcts, it is a far more variable technique resulting in differential damage to the myocardium alongside spontaneous restoration of LVEF that may complicate the assessment of regenerative cardiac patches [88]. Nevertheless, upon attaining a patch that achieves both robust retention of hPSC-CMs and restoration of post-MI LVEF in the permanent LAD ligation model, this patch should also be evaluated in other models including IRI and transverse aortic constriction-mediated HFrEF models.

## 5 Conclusion

This study demonstrates, for the first time, that MEW of high M_w_ polymers, in this case the MCL-PHA, P(3HO-*co*-3HD), is feasible and allows for the reproducible fabrication of intricate scaffolds with high fidelity. The MEW-PHA scaffolds demonstrated high biocompatibility in vitro, promoting structural maturation and functional improvement of hPSC-CMs, an outcome exploitable for robust in vitro cardiac drug testing. In vivo studies confirmed the MEW-PHA scaffolds were not surrounded by a fibrous capsule, often encountered when exogenous biomaterials are introduced in vivo, further confirming their excellent biocompatibility. Additionally, vascular regeneration of the infarct was achieved by the patches seeded with hPSC-CMVECs. However, despite this, the current iteration of MEW-PHA-based cardiac patches did not aid the retention of hPSC-CMs within the patch or prevent global cardiac functional decline following MI. Thus, the retention of hPSC-CMs and restoration of contractile function appears to be of greater importance to preventing post-MI cardiac functional decline than the restoration of the vasculature. Further insight into the timing of exogenous cell loss from the patch, whether due to immediate necrosis post-administration or gradual detachment, will be crucial to the future development of these MEW-PHA cardiac patches. This will firstly involve the use of a pre-vascularised patch in conjunction with either enhanced bioresorption kinetics or structurally immature hPSC-CMs that are more robust to hypoxic conditions and could then undergo in situ maturation upon the scaffold. These modifications will aim to enhance hPSC-CM retention and subsequent integration within the infarcted LV to develop a superior CTE solution that attenuates progression towards HFrEF.

## CRediT authorship contribution statement

**Qasim A. Majid:** Writing – review & editing, Writing – original draft, Visualization, Validation, Project administration, Methodology, Investigation, Formal analysis, Data curation, Conceptualization. **Pragati Pandey:** Writing – review & editing, Formal analysis, Data curation. **Mohamed Bellahcene:** Writing – review & editing, Formal analysis, Data curation. **Christopher L. Grigsby:** Writing – review & editing, Data curation. **Molly M. Stevens:** Writing – review & editing, Resources, Methodology. **Virpi Talman:** Writing – review & editing, Funding acquisition. **Daniel J. Stuckey:** Writing – review & editing, Formal analysis, Data curation. **Sian E. Harding:** Writing – review & editing, Supervision, Resources, Project administration, Funding acquisition, Conceptualization. **Ipsita Roy:** Writing – review & editing, Supervision, Project administration, Funding acquisition, Conceptualization. **Gábor Földes:** Writing – review & editing, Supervision, Project administration, Funding acquisition, Conceptualization.

## Funding

The research was funded by the 10.13039/501100000274British Heart Foundation Centre for Regenerative Medicine (RM/17/1/33377 and RE/13/4/30184) (all authors). Q.A.M. and V.T. attained further support from the Research Council of Finland (projects 321564, 353109; 328909), the 10.13039/501100005633Finnish Foundation for Cardiovascular Research, and the Sigrid Juselius Foundation. Additional funding was provided by the Higher Education Institutional Excellence Programme of the 10.13039/501100005881Ministry of Human Capacities in Hungary (G.F.) andthe Hungarian National Research Development and Innovation Fund (RRF-2.3.1-21-2022-00003, TKP2021-EGA-23) (G.F.); HyMedPoly (Grant agreement ID: 643050) (I.R.); 10.13039/501100000266Engineering and Physical Sciences Research Council (10.13039/501100000266EPSRC) (EP/V012126/1, EP/X021440/1; EP/X026108/1) (I.R.); StratNeuro (C.L.G.); the Whitaker International Programme (C.L.G.); the 10.13039/501100004359Swedish Research Council (10.13039/501100004359VR 4–478/2016) (C.L.G., M.M.S.); the 10.13039/501100019326UK Regenerative Medicine Platform “Acellular/Smart Materials – 3D Architecture” (MR/R015651/1) (M.M.S.); the 10.13039/501100000274British Heart Foundation Intermediate and Senior Basic Science Research Fellowships (FS/15/33/31608, FS/SBSRF/21/31020) (D.J.S.); the Medical Research Council (10.13039/501100000265MRC) (MR/R026416/1) (D.J.S.); and the 10.13039/100010269Wellcome Trust (212937/Z/18/Z) (D.J.S.).

## Declaration of competing interest

The authors declare that they have no known competing financial interests or personal relationships that could have appeared to influence the work reported in this paper.

G.F. and P.P. are currently employed by AstraZeneca and RxCelerate, respectively. The research outlined in this study was conducted during their employment at Imperial College London and is independent of their current roles. M.M.S. has invested in, consults for (or is on scientific advisory boards or boards of directors) and conducts sponsored research funded by companies related to the biomaterials field; has filed patent applications related to biomaterials; and has co-founded companies in the biomaterials field. I.R. is one of the directors of PHAsT Limited that develops biomedical-grade polyhydroxyalkanoates (PHAs) for a variety of applications, including healthcare and biomedical applications.

## Data Availability

Data will be made available on request.
