# Supplementary material for: Melt electrowritten medium chain length polyhydroxyalkanoate cardiac patches for Post-MI cardiac regeneration
Source: Mater Today Bio. 2025 Aug 29;34:102256. doi: 10.1016/j.mtbio.2025.102256 (PMC12446554; doi:10.1016/j.mtbio.2025.102256)
Supplement: Multimedia component 1 [file mmc1.pdf]

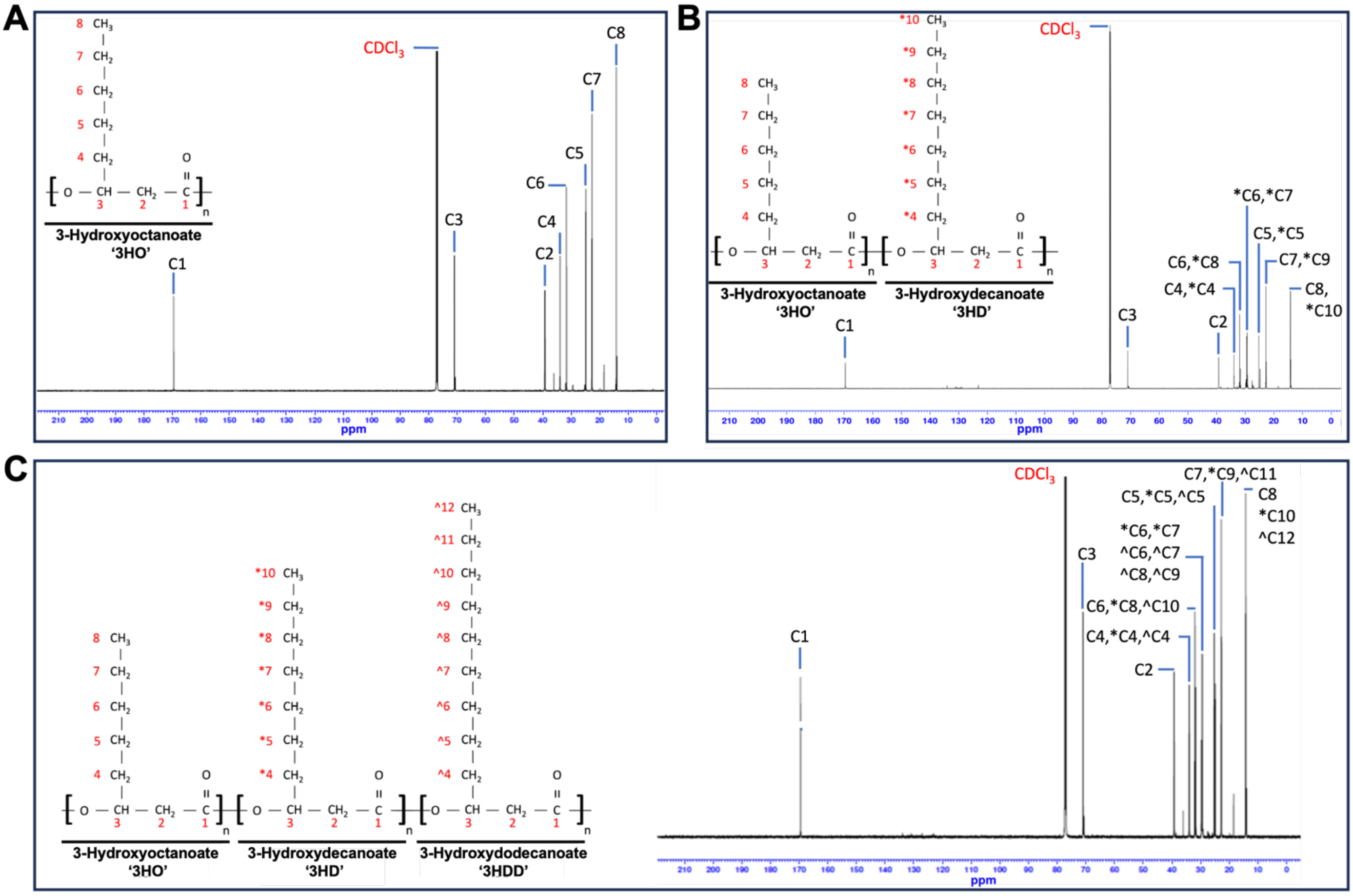

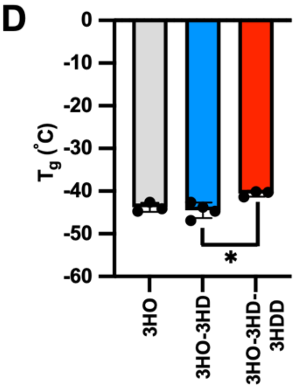

E

MCL-PHA strip	σ_u (MPa)	E (MPa)	ε_b (%)
**P(3HO)**	5.19 ± 0.44	3.62 ± 0.66	345.98 ± 38.88
**P(3HO-*co*-3HD)**	10.10 ± 3.58	8.84 ± 2.20	410.23 ± 95.63
**P(3HO-*co*-3HD-*co*-3HDD)**	3.45 ± 1.02	1.39 ± 0.30	302.20 ± 67.98

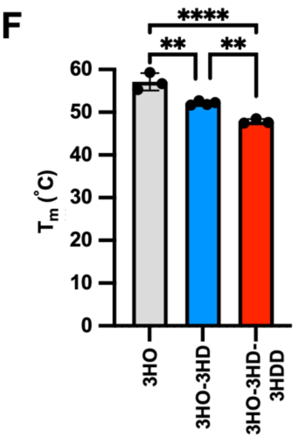

Fig. S1. Carbon-13 NMR spectra revealing the monomeric structures of (**A**) poly(3-hydoxyoctanoate), (**B**) poly(3-hydroxyoctanoate-*co*-3-hydroxydecanoate), and (**C**) poly(3-hydroxyoctanoate-*co*-3-hydroxydecanoate-*co*-3-hydroxydodecanoate). The structural formula of each MCL-PHA is displayed within the respective spectrum. * refers to the carbon atoms present in the 3-hydroxydecanoate monomer and ^ denotes the carbons atoms present in the 3-hydroxydodecanoate monomer. (**D**) The glass transition temperature (T_g) of P(3HO) (grey bar), P(3HO-*co*-3HD) (blue bar), and P(3HO-*co*-3HD-*co*-3HDD) (red bar). Mean ± S.D., N=3 whereby three DSC curves were analysed for each MCL-PHA. One-way ANOVA with Bonferroni's post-hoc test: * $P<0.05$. (**E**) Calculated apparent-ultimate tensile strength (σ_u), -elastic modulus (E), and -elongation at break (ε_b) of solvent cast-strips derived from the three MCL-PHAs. Mean ± S.D., N=4-6 separate strips. (**F**) The melting temperature (T_m) of P(3HO) (grey bar), P(3HO-*co*-3HD) (blue bar), and P(3HO-*co*-3HD-*co*-3HDD) (red bar). Mean ± S.D., N=3 whereby three DSC curves were analysed for each MCL-PHA. One-way ANOVA with Bonferroni's post-hoc test: ** $P<0.01$ **** $P<0.0001$.

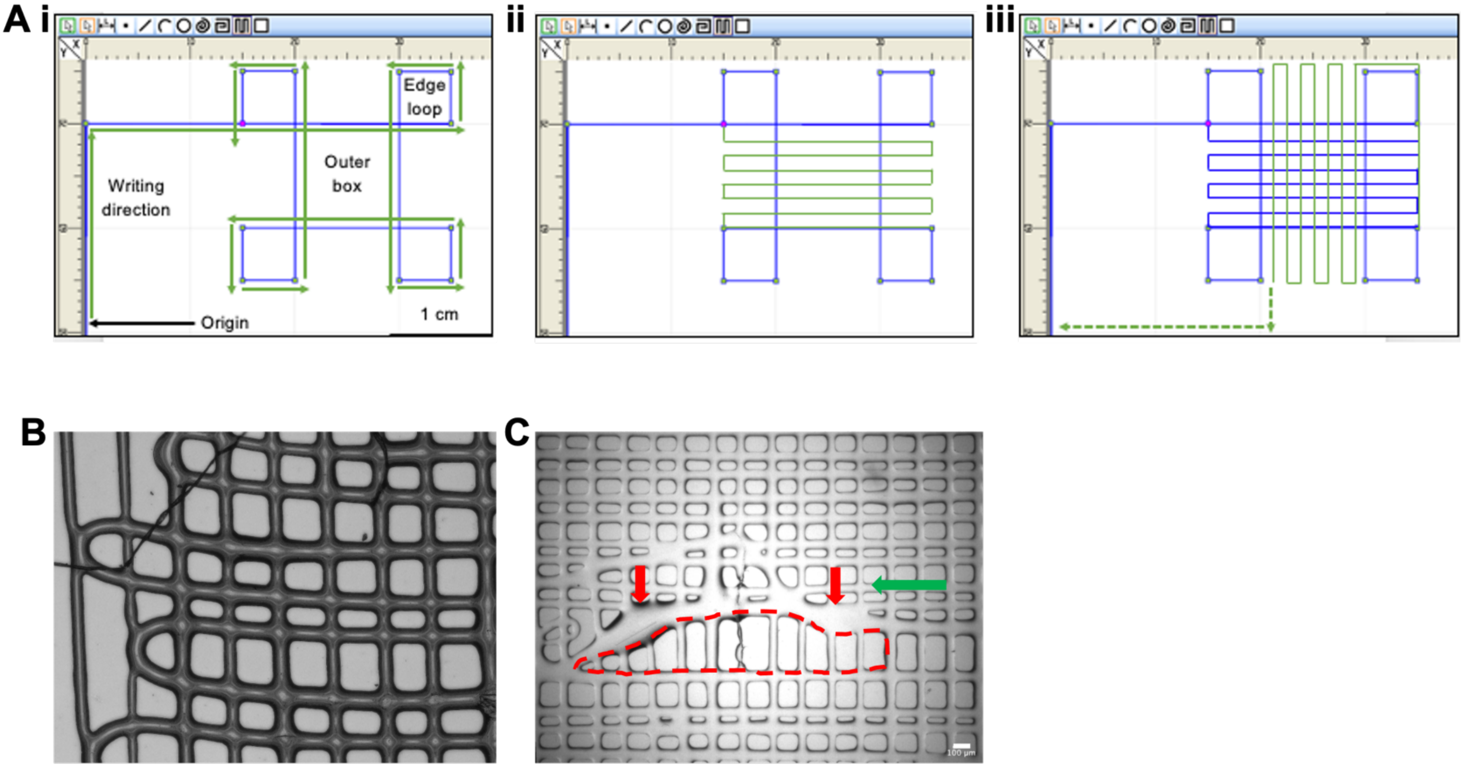

D

	Intended dimensions (µm)				Calculated dimensions (µm)			
Scaffold	Length	Width	Diagonal Length	Aspect ratio	Length	Width	Diagonal Length	Aspect ratio
A	100	150	180.28	01:01.5	104.85 ± 6.07	140.54 ± 2.42	176.63 ± 2.91	1:1.35 ± 0.12
B	100	200	223.61	01:02	106.53 ± 8.96	144.10 ± 2.61	180.12 ± 3.71	1:1.36 ± 0.16

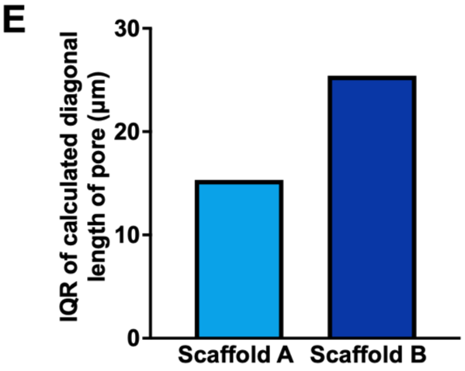

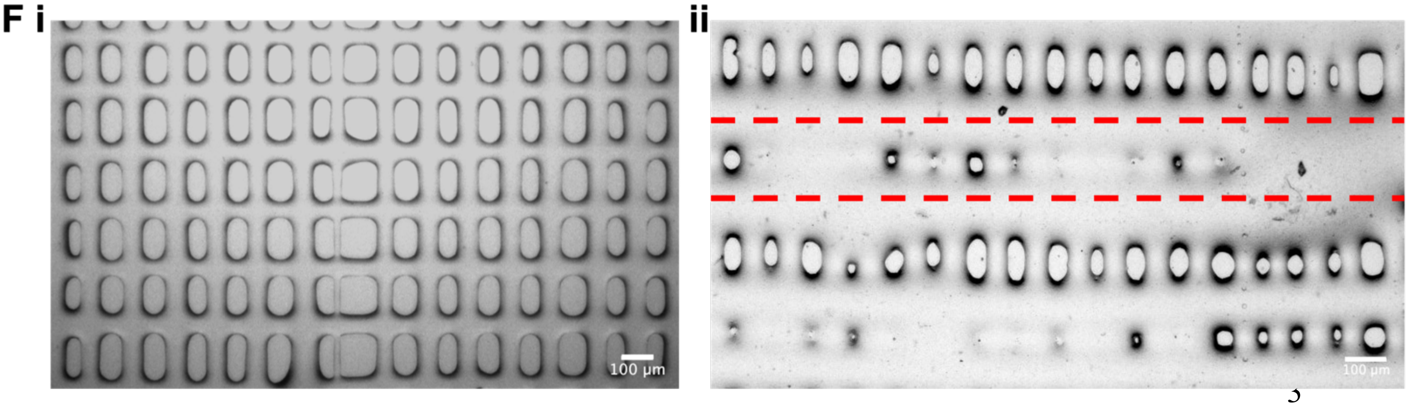

Fig. S2. (**A**) Schematic illustration of the MEW-PHA scaffold design within the SEL programme: (**i**) The computer aided design (CAD, blue lines) stipulates an initial 1 cm^2 outer box from which edge loops extend. The writing direction relative to the origin (black arrow) is indicated by the green arrows. Scale bar represents 1 cm. (**ii**) Fibres are then deposited in the χ axis followed by the (**iii**) γ axis after which the jet is designed to return to the origin (dotted green line). Representative images of the (**B**) heterogenous fibre deposition at the edge of the scaffolds outer box and (**C**) the local perturbations (dotted red line) observed within initial MEW-PHA scaffolds. The green arrow indicates the direction of MEW, and the red arrows highlight the deposition of large volumes of polymer. Scale bar represents 100 µm. (**D**) Summary table displaying the intended and calculated dimensions (length, width, diagonal length, and aspect ratio) of pores within porous MEW-PHA scaffolds A and B. Mean ± S.D., N=3. (**E**) The interquartile range (IQR) of the calculated diagonal length of pores within scaffold A and B. (**F**) Representative images of (**i**) single- and (**ii**) multi-layer MEW-PHA scaffolds derived from the same initial CAD file resulting in the merging of neighbouring fibres and loss of pores (dotted red lines) following MEW layering. Scale bars represent 100 µm.

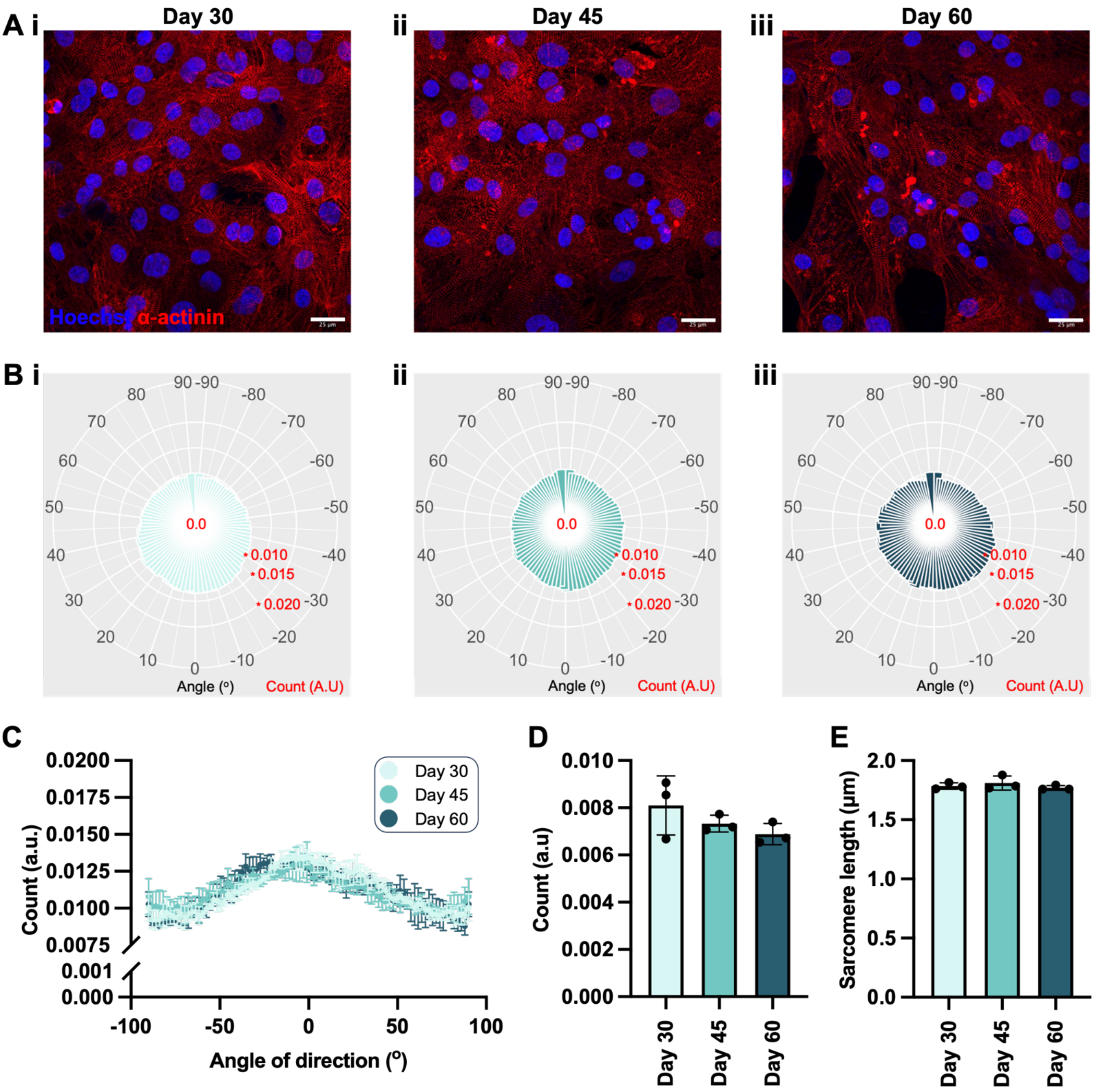

Fig. S3. (**A**) Representative immunofluorescent images of day 15 hPSC-CMs cultured to (**i**) day 30, (**ii**) day 45, and (**iii**) day 60 on TCP. Cells were stained for the cardiomyocyte marker α-actinin (red) and the nuclear marker Hoechst (blue). Scale bars represent 25 μm. (**B**) Rose diagram visualisation of hPSC-CM sarcomere alignment following culture on TCP to (**i**) day 30, (**ii**) day 45, and (**iii**) day 60. Quantification of (**C**) sarcomere alignment by way of the (**D**) count value at half maximum. (**E**) Quantification of sarcomere length. (**D-E**) Mean ± S.D., N=3. One-way ANOVA with Bonferroni's post-hoc test revealed no significant difference.

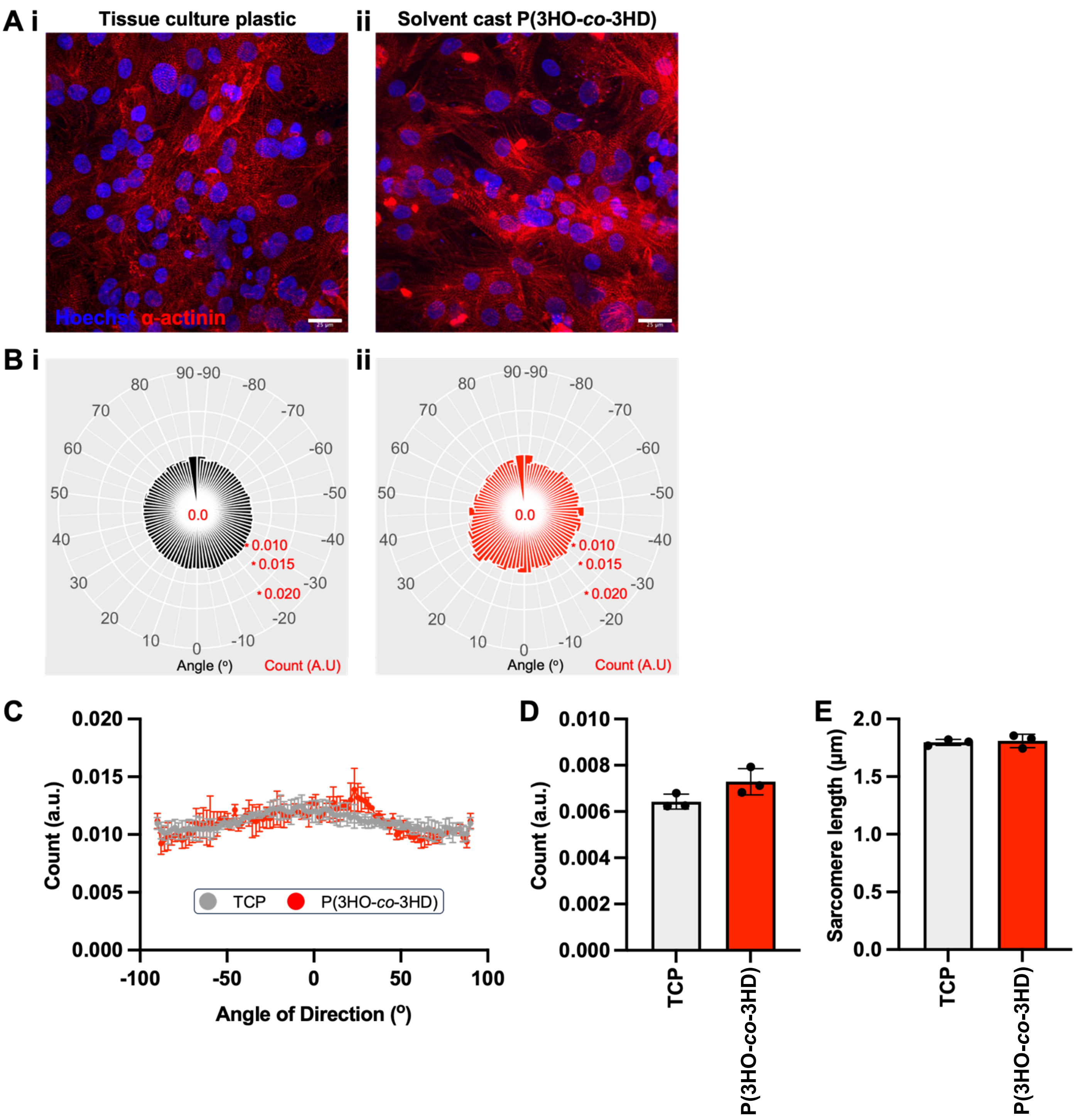

Fig. S4. (**A**) Representative immunofluorescent images of hPSC-CMs cultured on (**i**) TCP and (**ii**) P(3HO-*co*-3HD)-derived solvent cast films for one-week. Cells were stained for the cardiomyocyte marker α-actinin (red) and the nuclear marker Hoechst (blue). Scale bars represent 25 µm. (**B**) Rose diagram visualisation of hPSC-CM sarcomere alignment following culture on (**i**) TCP and (**ii**) P(3HO-*co*-3HD)-derived solvent cast films. Quantification of (**C**) sarcomere alignment, by way of the (**D**) count value at half maximum. (**E**) Quantification of sarcomere length. (**D-E**) Mean ± S.D., N=3. Unpaired two-tailed t-test revealed no significant difference.

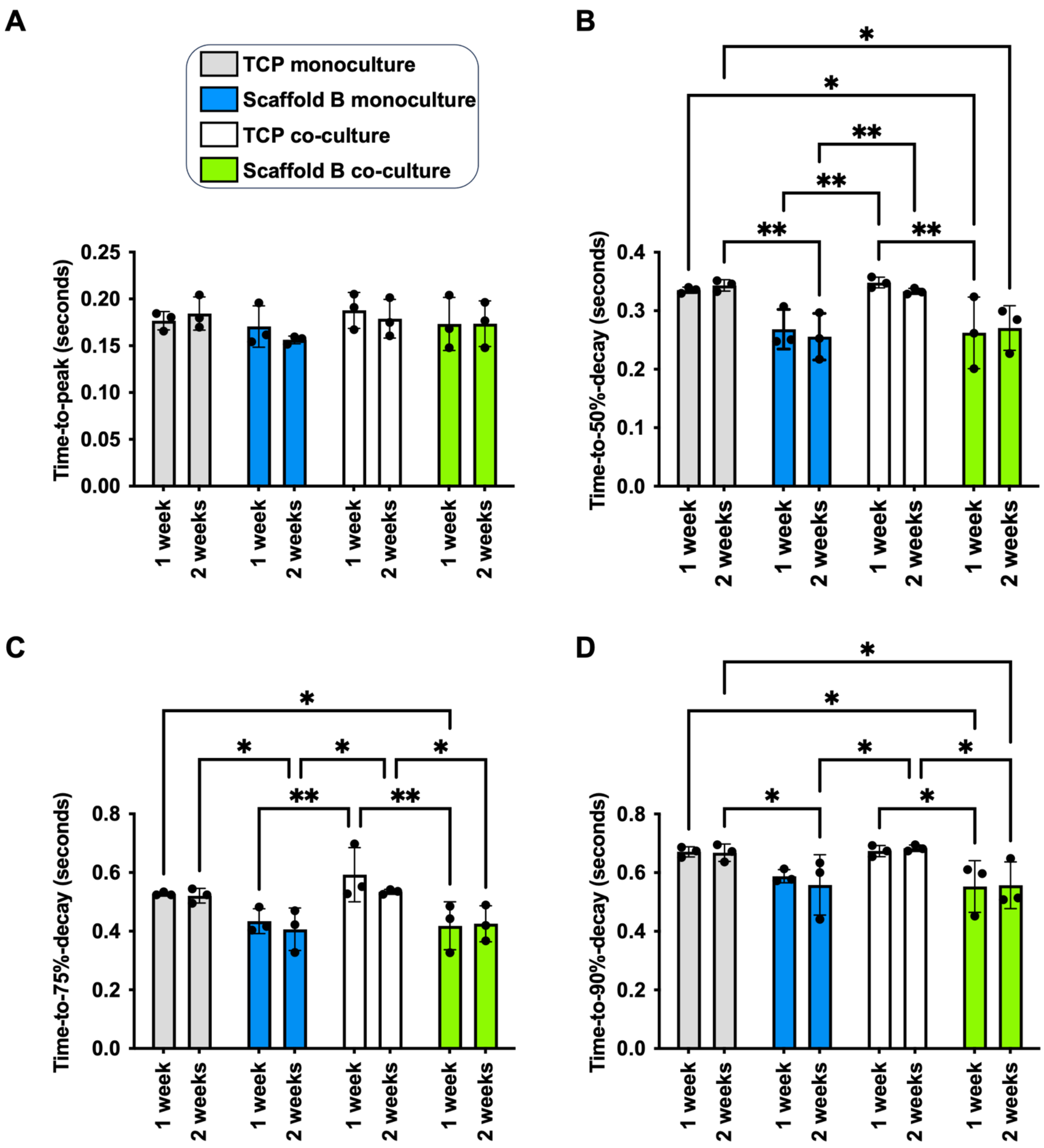

Fig. S5. Ca^{2+} handling kinetics of hPSC-CM monocultures seeded on TCP (grey bars) or scaffold B (blue bars) or co-cultures of hPSC-CMs, and hPSC-CMVECs seeded on TCP (white bars) or scaffold B (green bars) for one or two weeks: (**A**) Time-to-peak Ca^{2+} transient, time-to- (**B**) 50%-, (**C**) 75%-, and (**D**) 90%-decay of Ca^{2+} transient. Mean ± S.D., N=3. Two-way ANOVA: * $P<0.05$, ** $P<0.01$.

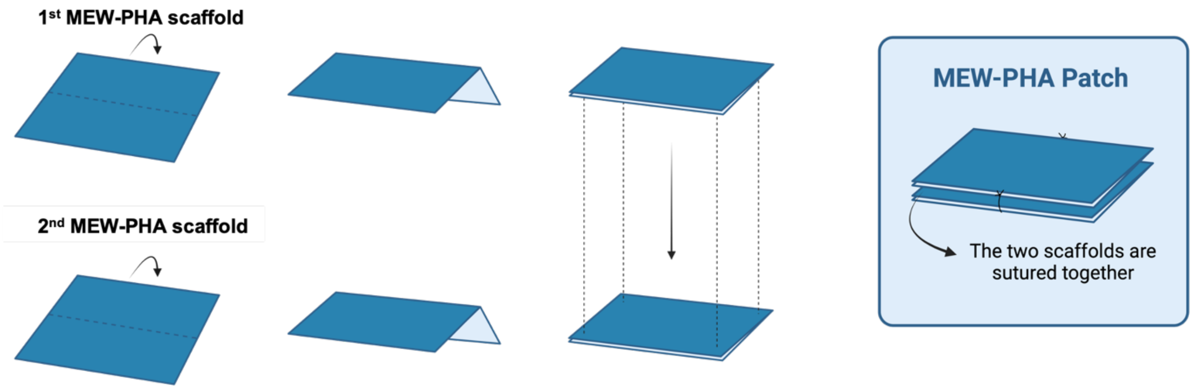

Fig. S6. Graphical illustration of the folding and stacking of scaffold B to attain the final MEW-PHA cardiac patches. The darker side of the scaffold illustrates the cell-seeded side.

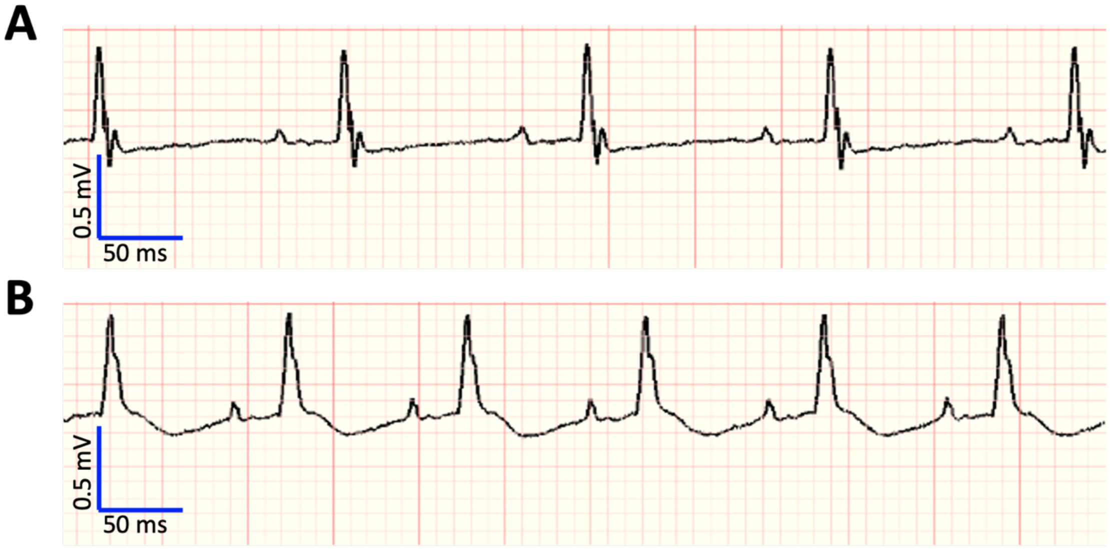

Fig. S7. Representative trace acquired from the three-lead ECG of NSG mice (**A**) at the start of the surgical procedure and (**B**) one minute following LAD ligation.

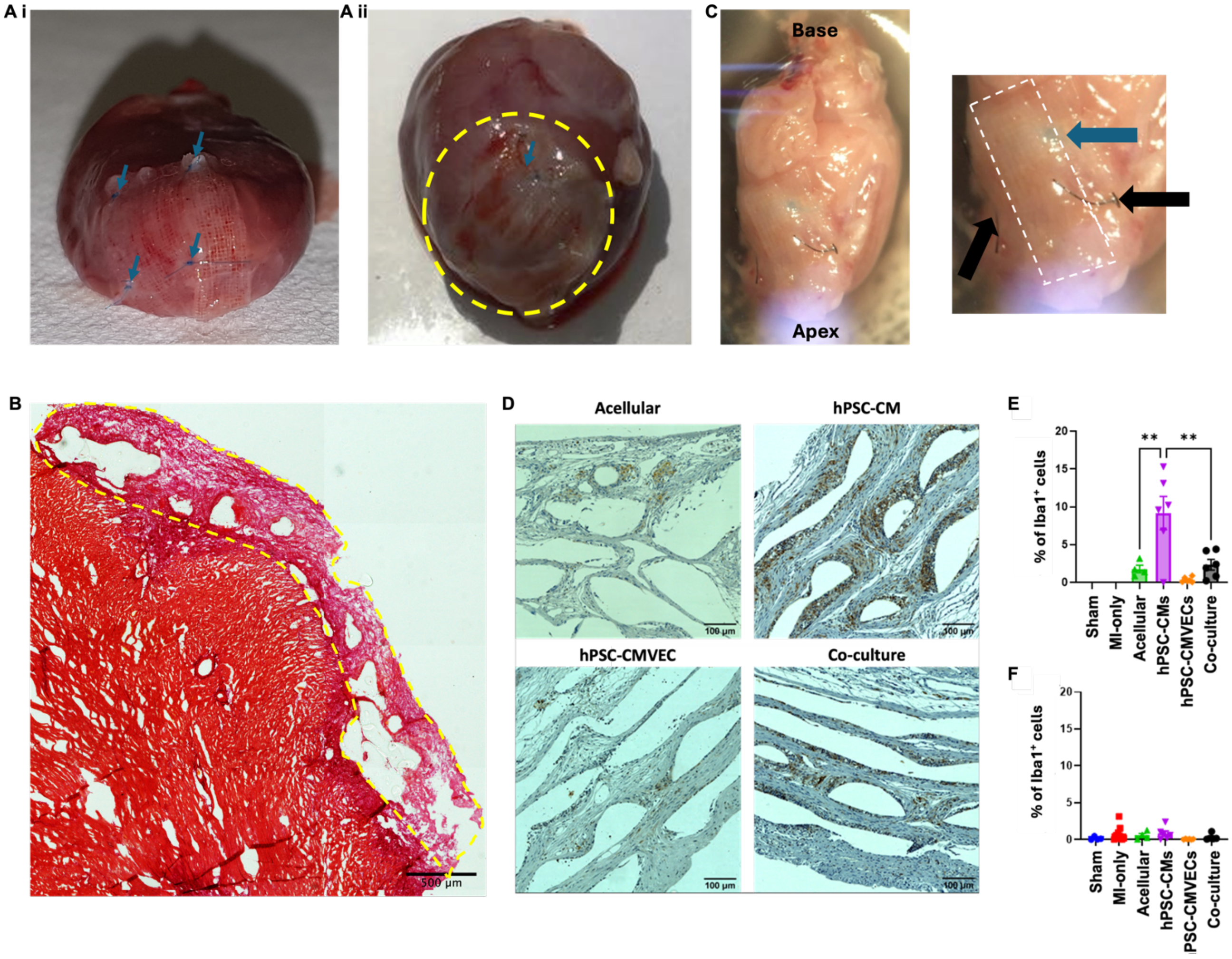

Fig. S8. (A) Macroscopic image of a single acellular MEW-PHA scaffold placed onto the LV of a healthy Sprague Dawley rat for (i) 24 hours and (ii) 7 days. The sutures used to adhere the scaffold to the rat LV are highlighted by the blue arrow and the yellow circle demarcates the patch. **(B)** Picrosirius red staining of the rat LV and acellular MEW-PHA patch 7 days post-implantation. The patch is delineated by the yellow dotted line. Scale bar represents 500 µm. **(C)** Macroscopic image of the cardiac patch placed onto the LV of a NSG mouse for one month. Inset: The white dashed box highlights the patch; the black arrows denote the sutures used to hold the two scaffolds in place within the patch, and the blue arrow indicates the suture used to ligate the LAD. **(D)** Representative DAB images of the different MEW-PHA-based cardiac patches four-weeks post-administration to the NSG mouse LV. Samples were stained for the macrophage marker Iba1 (brown). Scale bars represent 100 µm. Quantification of the percentage of Iba1-positive cells within **(E)** the different patches and **(F)** the proximal healthy myocardium of the different groups. Mean ± SEM. N=4-9. One-way ANOVA: ** P<0.01
